# Solving disorder in (3D) real space: a comparative study of the three-dimensional difference pair distribution function and atomic resolution holography reconstructions

**DOI:** 10.1107/S1600576725005977

**Published:** 2025-08-08

**Authors:** Jens R. Stellhorn, Arianna Minelli, Emily G. Meekel, Ella M. Schmidt

**Affiliations:** a Co-Creation Institute for Advanced Materials, Shimane University, Shimane690-8504, Japan; b Neutron Scattering Division Oak Ridge National Laboratory,Oak Ridge TN 37831 USA; c Institute for Integrated Cell-Material Science, Kyoto University, Kyoto606-8501, Japan; d Faculty of Geosciences, MARUM and MAPEX, University of Bremen, Bremen, Germany; University of Silesia in Katowice, Poland

**Keywords:** diffuse scattering, three-dimensional difference pair distribution function analysis, 3D-ΔPDF, atomic resolution holography, disorder

## Abstract

The quantitative analysis of local ordering principles in disordered crystalline systems has gained much attention over the past few years, as it is often considered crucial for optimizing material functionality. This development has been driven by significant advancements in computational and experimental methods, which have led to the establishment and widespread use of various analytical techniques. In this study, we perform model calculations to compare the effectiveness of atomic resolution holography and three-dimensional difference pair distribution function analysis (3D-ΔPDF). Using Cu_3_Au as a model system, we demonstrate an approach to derive local order parameters quantitatively and show that both techniques are well suited to quantifying chemical short-range order correlations and local bond-distance variations. By evaluating the strengths and limitations of both techniques, we advocate for their combined use to solve complex short-range order problems accurately.

## Abstract 2

This study uses computational models to compare the three-dimensional difference pair distribution function and atomic resolution holography for analyzing three-dimensional disorder, revealing their complementary strengths in quantitative pair correlation analysis versus chemical specificity and advocating for a combined approach to tackle complex materials.

## 1. Introduction

Uniquely positioned between ordered crystals and amorphous solids, disordered crystalline materials have emerged as an efficient platform for the discovery and optimization of unprecedented functionalities. Notable examples include enhanced thermoelectric properties observed in doped PbTe (Fu *et al.*, 2017 ▸), ionic conductivity in disordered solid-state batteries (Martinez de Irujo-Labalde *et al.*, 2024 ▸), ion migration in optoelectronic materials such as halide perovskites (Weadock *et al.*, 2023 ▸; Dubajic *et al.*, 2025 ▸), spin-glass behavior in dilute AuFe and CuMn alloys (Kawamura & Taniguchi, 2015 ▸), and the remarkable ferro- and piezo-electric properties of the relaxor ferroelectric PbMg_1/3_Nb_2/3_O_3_ (Bokov, 1997 ▸).

In most of these cases, and many others, the disorder is governed by short-range interactions between structural components, resulting in correlated disorder. Optimizing the functionality of such locally ordered crystalline materials requires the identification and understanding of the local rules that dictate their structure, ultimately enabling precise manipulation and control of their properties.

Various techniques are available to investigate the local structure of correlated disordered materials. Total scattering methods, such as diffuse scattering (DS), are particularly effective in revealing correlations between disordered degrees of freedom (Keen & Goodwin, 2015 ▸). Alternatively, extended X-ray absorption fine structure (EXAFS) provides detailed information on local atomic environments (O’Day *et al.*, 1994 ▸), whereas resonant diffraction probes the chemical and short-range order of materials through absorption while maintaining the long-range order sensitivity of diffraction techniques (Hodeau *et al.*, 2001 ▸; Yasui *et al.*, 2023 ▸; Waseda, 1984 ▸; Dmitrienko & Ovchinnikova, 2000 ▸). Solid-state NMR offers insights into local environments for specific active nuclei (Moran *et al.*, 2017 ▸; Yasui *et al.*, 2023 ▸), and advanced spectroscopic techniques, such as Raman (Pimenta *et al.*, 2007 ▸) and infrared (IR) (Sapnik *et al.*, 2018 ▸) spectroscopy, provide an indirect view of disordered structures. While these methods often yield qualitative insights, achieving an accurate quantitative characterization of the disorder remains a significant challenge.

Quantifying structural (correlated) disorder in crystalline materials typically involves comparing experimental data with models that accurately capture atomic correlations. Real-space Monte Carlo simulations are commonly used to generate such models, employing experimentally derived local rules to minimize the configurational energy. However, despite their utility, Monte Carlo methods face challenges, such as the need to sample vast configurational spaces and the risk of becoming trapped in a local minimum. Moreover, the accuracy of these models heavily depends on the completeness and accuracy of the input data, which experimental techniques often struggle to provide.

A solution to these challenges is to use experimental techniques capable of extracting quantitative real-space atomic correlations for disordered crystalline systems. Two such approaches are the 3D difference pair distribution function (3D-ΔPDF) – derived from DS – and atomic resolution holography (ARH) (Stellhorn *et al.*, 2024 ▸). Specifically, both techniques use the Patterson space to provide insight into disorder at the atomic level.

The 3D-ΔPDF is obtained by isolating the DS signal from single-crystal total scattering data and performing a Fourier transform (FT). The output FT reveals deviations from the average structure, capturing atomic correlations and local structural distortions in three-dimensional space (Weber & Simonov, 2012 ▸). ARH, in contrast, uses interference patterns generated by characteristic X-ray fluorescence scattering to extract directly the local atomic environment around specific elements. The resulting holograms can be transformed using an FT-like algorithm to yield the 3D environment of electron density around the target element, which can be regarded as an element-specific 3D PDF.

Both DS and ARH have been used to study diverse types of disorder in a wide range of materials. For example, *chemical disorder*, *e.g.* doped CdTe (Nagaoka *et al.*, 2023 ▸) and Cu_3_Au (Schweika *et al.*, 2000 ▸; Dąbrowski *et al.*, 2015 ▸); *displacement disorder*, *e.g.* framework distortions in the zeotypic material AlPO_4_ (Withers & Liu, 2005 ▸); or a combination of the two, *e.g.* the effect of heavy element doping in Fe_2_VAl Heusler-type thermoelectric alloys (Kimura *et al.*, 2020 ▸), the location of vacancies of the carbonate molecule in Ba_3_Co_2_O_6_(CO_3_)_0.7_ (Morgan *et al.*, 2021 ▸; Igarashi *et al.*, 2012 ▸), and the oxygen vacancies and subsequent relaxation on yttria- and calcia-stabilized zirconia (Proffen *et al.*, 1996 ▸; Welberry *et al.*, 1992 ▸; Schmidt *et al.*, 2023 ▸; García-Martín *et al.*, 2008 ▸). DS has also been used to investigate the rearrangement of structures, such as the structural relaxation arising from dopants (Gutmann *et al.*, 2024 ▸), the formation of complex polarization textures in the ferroelectric PbTiO_3_ (Zatterin *et al.*, 2024 ▸) and many other cases (Welberry & Weber, 2016 ▸). Meanwhile, ARH is particularly effective in identifying specific dopant sites, as demonstrated for the topological insulator Mn:Bi_2_Te_3_ (Hosokawa *et al.*, 2017 ▸), Co-doped TiO_2_ for spintronic applications (Hu *et al.*, 2015 ▸) and the scintillator Nd:LaF_3_ (Stellhorn *et al.*, 2020 ▸).

Despite their successes, both techniques face challenges in routine quantitative analysis, with limited software tools available for the systematic refinement or modeling of disorder. Quantitative analysis of DS often relies on special­ized programs such as *Yell* (Simonov *et al.*, 2014*b* ▸), and using *NexPy* (Kienzle *et al.*, 2024 ▸; Krogstad *et al.*, 2020 ▸) for 3D-ΔPDF analysis, *DISCUS* (Neder & Proffen, 2008 ▸) for direct Monte Carlo simulations and *rmc-discord* (Morgan *et al.*, 2021 ▸) for reverse Monte Carlo simulations. Notably, these tools remain complex and limited in scope. ARH still lacks dedicated software for the quantitative treatment of disorder, with most studies relying on the derivation of a qualitative model.

In the case of DS analysis, several parameters that describe pair correlations have been defined in the past. The most common is probably the Warren–Cowley short-range order parameter (Warren *et al.*, 1951 ▸), 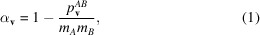
where *m*_*A*_ and *m*_*B*_ are the average occupancies of atoms of type *A* and type *B*, respectively, and 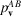 is the conditional probability of finding an atom of type *A* at an interatomic vector **v** from an atom of type *B*. Similarly, correlation parameters such as *C*_*lmn*_ (Welberry & Weber, 2016 ▸) or *c*_*uvw*_ (Simonov *et al.*, 2014*a* ▸) have been used to give a quantitative description of chemical short-range order. Despite the small differences in notation, these parameters all essentially quantify deviations in the probability of finding specific atomic pairs, as opposed to a completely random structure. This difference is directly encoded in the experimentally observed DS. The 3D-ΔPDF then directly maps these deviations from the average structure in Patterson space.

Thus, while DS and ARH differ fundamentally in their experimental methodologies and analytical approaches, the two techniques offer complementary perspectives on structural disorder by probing correlations in 3D real space. By advancing their routine quantitative analysis, we can reveal deeper insights into the complex behavior of correlated disordered materials, paving the way for enhanced functionality in material design.

**Figure 1:**
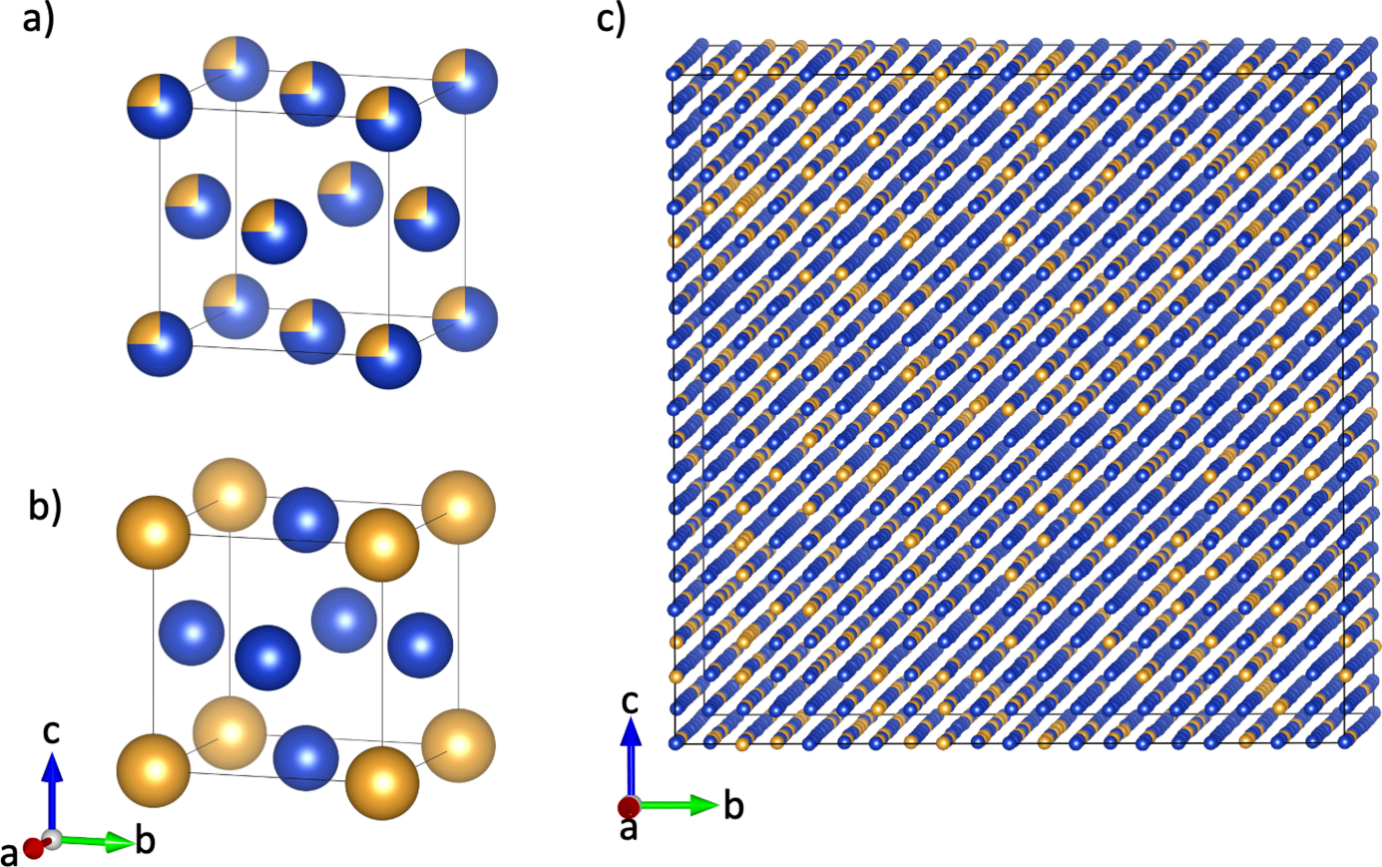
(*a*) Average disordered structure of Cu_3_Au. (*b*) Average structure of an ordered *L*1_2_ unit cell. (*c*) 10 × 10 × 10 supercell of Cu_3_Au with disordered distribution of Cu and Au. Cu in blue and Au in gold. Figures created using *VESTA* (Momma & Izumi, 2011 ▸).

In this work, we performed a computational study with the alloy Cu_3_Au (Fig. 1 ▸) as a model system to demonstrate the application of ARH and DS via 3D-ΔPDF analysis to assess structural disorder in a representative binary system. This combined approach offers a comprehensive quantitative analysis of the strengths and weaknesses of the two methods when identifying short-range order. For simplicity, we limit the model study to the toy model of Cu_3_Au, but the results are not limited to this model system. The methodology as we demonstrate it here can be transferred to other binary and more complex disordered systems to identify and quantify disorder. We will close this contribution by commenting on the accessibility of the two methods and discussing potential more complex use cases in systems with more than two disordered components.

## 2. Model system and data generation

### 2.1. Generation of disordered structures

In our study, we use the alloy Cu_3_Au as a model system, which has a face-centered cubic structure (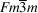, *a* = 3.74 Å). We examine a total of ten different model structures: five with varying levels of chemical short-range order (CSRO), three with different degrees of interatomic distance relaxation and random chemical order, and two with a combination of CSRO and size-effect-like relaxations. In the text, they are defined as CSRO, ‘Size’ and ‘Combined’, respectively, and Table 1 ▸ provides a brief description of each structure. These structures are generated using the *DISCUS* program. Each structure is a 10 × 10 × 10 supercell, starting with a total of 4000 Cu atoms.

To introduce the disorder, we first randomly replace 25% of the Cu atoms with Au atoms. For structures with random chemical order (CSRO0, Size0.01, Size0.05, Size0.10), the site occupancies are not further manipulated. For the other structures (CSRO+0.3, CSRO+0.15, CSRO−0.15, CSRO−0.3, Combined+0.15 and Combined−0.15), we use Monte Carlo simulations in *DISCUS* to achieve the desired levels of CSRO. We use the Warren–Cowley short-range order parameter α [see equation (1)] to guide the Monte Carlo simulations and measure the level of CSRO achieved. More details on the Monte Carlo simulation are provided in the supporting information. A representative superstructure with chemical short-range order is shown in Fig. 1 ▸(*c*).

For structures where atomic positions are relaxed, we again use Monte Carlo simulations in *DISCUS* (Neder & Proffen, 2008 ▸). First, to introduce some randomness in the atomic positions, all atoms are displaced by a small randomly chosen vector that mimics thermal vibrations (with *B* = 2.0 Å^2^). During the simulations, the atoms are then shifted from their initial positions, with spring-like forces used to control the distances between neighboring atoms. Specifically, the nearest-neighbor distances 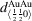 for Au–Au pairs, 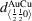 for Au–Cu pairs and 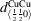 for Cu–Cu pairs are adjusted. 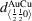 is kept at 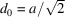, while 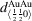 is increased by a factor of (1 + δ) (where δ = 0.01, 0.05 and 0.1 in the structures Size0.01, Size0.05 and Size0.10, respectively) and 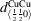 is decreased by a factor of (1 − δ/9).

For the two structures that combine CSRO and size-effect relaxations, the distance relaxations with δ = 0.1 were applied to the chemically sorted structures CSRO±0.15.

### 2.2. Diffuse scattering and 3D-ΔPDF calculation

**Figure 2:**
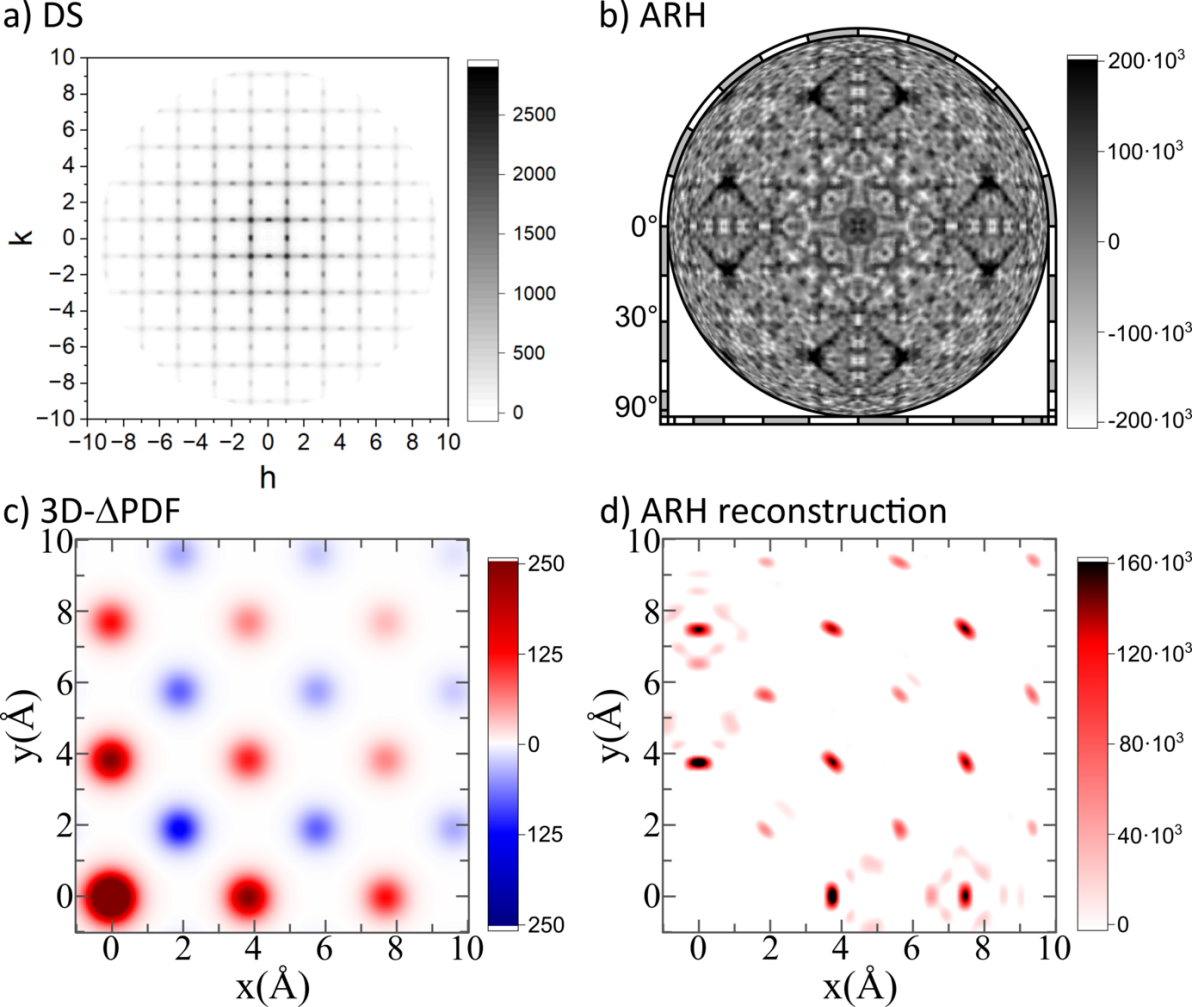
Computed data for the CSRO−0.3 structure. (*a*) Calculated diffuse scattering in the *hk0* layer. (*b*) ARH hologram. (*c*) 3D-ΔPDF in the *xy*0 layer. (*d*) Real-space reconstruction of ARH in the *xy*0 layer.

The diffuse scattering of the generated structures was calculated using the *DISCUS* program. To obtain high-quality diffuse scattering patterns despite the relatively small model crystal, we utilized a variation of the *lots* algorithm (Neder & Proffen, 2008 ▸; Proffen & Welberry, 1997 ▸; Welberry & Proffen, 1998 ▸), which ensures that every possible 5 × 5 × 5 subcell of the model structure supercell is probed. The diffuse scattering, excluding Bragg reflections, was calculated on a three-dimensional grid in the ranges −10 ≤ *h*, *k*, *l* ≤ 10 with a step size of Δ*h* = Δ*k* = Δ*l* = 0.1. We used X-ray atomic form factors with a wavelength of λ = 0.71 Å and no dispersion correction was applied. An example of the diffuse scattering in the *hk*0 layer of the CSRO−0.3 structure is shown in Fig. 2 ▸(*a*).

The resulting diffuse scattering data were further processed by symmetry averaging for 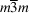 Laue symmetry using the program *Meerkat* (Simonov, 2020 ▸). The 3D-ΔPDF, illustrated in Fig. 2 ▸(*c*), was obtained by performing a Fourier transformation of the diffuse scattering data.

### 2.3. ARH calculation

To calculate the atomic resolution holograms of the generated structures, we used the *3D-Air-Image* program (Matsushita *et al.*, 2018 ▸). The holograms were calculated as follows. First, for every atom of a certain element in the large supercell model (called ‘emitter atoms’), we determined the local environment in a 25 Å radius. For all of these clusters, a hologram was calculated by a sum of elemental holograms formed by each neighboring atom (Matsushita *et al.*, 2018 ▸). We chose the Au atoms as emitter atoms and calculated the Au holograms in the energy range of 10.0–14.75 keV in steps of 250 eV. Similarly to the calculation of the DS data, no dispersion correction was applied here.

These holograms were subsequently used for the reconstruction of 3D real space by the *Barton* algorithm (Barton, 1988 ▸; Barton, 1991 ▸), which describes the FT-like algorithm to extract the real-space image function *U*(*r*) from the hologram χ(*k*) using a surface integral:
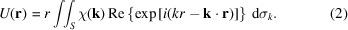
This function effectively describes the local environment (*i.e.* the distribution of electron density, in the case of X-ray holography) around a specific element (Au, in our case) and can be interpreted as an element-specific 3D pair distribution function. More information on the details of the procedure can be found elsewhere (Matsushita *et al.*, 2018 ▸).

### 2.4. 3D-ΔPDF analysis

For the quantitative analysis of our simulated diffuse scattering data, we used the program *Yell* (Simonov *et al.*, 2014*b* ▸; Simonov *et al.*, 2014*a* ▸). For data sets exhibiting only chemical short-range order, we refined one scale parameter and 37 occupational correlation parameters, which describe the probabilities of finding Au–Au pairs separated by the shortest interatomic vectors up to the 〈322〉 vector. All refinements converged normally, and the refined parameters with uncertainties, as derived from the least-squares refinement, are provided in the supporting information.

The analysis of data sets that include displacement disorder is more complex. For each interatomic vector considered, we refined one parameter for chemical short-range order, one parameter for size-effect relaxation and several parameters that describe atomic displacement correlations. To keep the number of refined parameters as low as possible, it was assumed that the interatomic distance relaxation is in the direction of the considered interatomic vector. However, several parameters are needed to describe the atomic displace­ment correlations, as this accounts for the fact that the nearest-neighbor vector distribution is much smaller than that of the average interatomic vector distribution, as determined by the average structure displacement parameter (ADP) (Weber & Simonov, 2012 ▸). In total, we refine 32 parameters: one overall scale parameter, one average structure isotropic displacement parameter, six chemical short-range order parameters, six size-effect parameters and 18 displacement pair-correlation parameters. Unlike the structures with pure chemical short-range order, we only consider correlations up to the 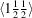 vector here to limit the number of refined parameters. Again, all refinements converged normally, and the refined parameters are provided in the supporting information.

### 2.5. ARH analysis

In contrast to the 3D-ΔPDF analysis, in the real-space reconstruction from ARH only the positive signals are meaningful. They represent the local environment around a specific target element (Au, in this case). The signal intensity χ_ARH_ is proportional to the number of atoms at a given interatomic vector as well as to the electron density (or, to a good approximation, the atomic number *Z*). In the simplest case of the real-space reconstruction of a small system from a single hologram, each signal follows a sinc(*r*) function with its main peak at the interatomic vector [see Matsushita *et al.* (2018 ▸), and equations regarding inverse mode holography therein]. However, when using multi-energy holograms (which is the state-of-the-art approach), the signals can be reasonably approximated by a Gaussian function, greatly simplifying the data analysis procedure.

For determining the chemical short-range order, the observed signals are integrated around the nominal inter­atomic vectors to yield χ_ARH_. As we observe a complex background variation, we normalize the observed integrated intensities by the values we obtain from the CSRO0 structure for the same interatomic vector. While this is straightforward for the simulated data we use here, we acknowledge that the treatment of real experimental data will be more difficult. As a strategy, we suggest calculating a hologram from a randomly disordered structure and subsequently refining a scale parameter to ‘far-away’ un-correlated interatomic vectors. With this approach, a quantification of the integrated intensities χ_ARH_ of the holograms should be viable.

To estimate the Warren–Cowley short-range order parameter α, as a first approximation we assume that the intensity in an ARH reconstruction at a certain interatomic vector **v** is given as the weighted (with the atomic number *Z*) sum of the probabilities of finding a neighboring element *i* around the central atom *K*: 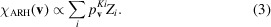
For the normalization signal, we assume that the probabilities correspond to a random distribution of neighbors. For our case of Cu_3_Au, 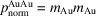 and 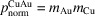. The relative area *R* is hence described as
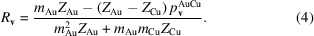
From equation (4) we can calculate an estimation of 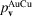 and hence of the Warren–Cowley short-range order parameter: 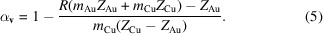
In the structures with displacement disorder, a Gaussian function was fitted to the ARH intensity along the interatomic vector direction. This Gaussian contains the contributions of each element pair (Au–Au and Au–Cu). To disentangle the different interatomic distances, we assume that the Au–Cu nearest-neighbor distance remains fixed at its average value of 2.641 Å as defined by the lattice constant.

## 3. Results and discussion

The aim of this study is to demonstrate that both ARH and the 3D-ΔPDF are well suited for analyzing disorder in three-dimensional systems. Using a binary model system, we illustrate the effectiveness of both methods. The results are presented in three parts: Section 3.1 focuses on structures exhibiting CSRO, while Section 3.2 examines structures with size-effect relaxations. Finally, Section 3.3 discusses arguably the most relevant case, where CSRO and size-effect relaxations co-exist.

### 3.1. Chemical short-range order

**Figure 3:**
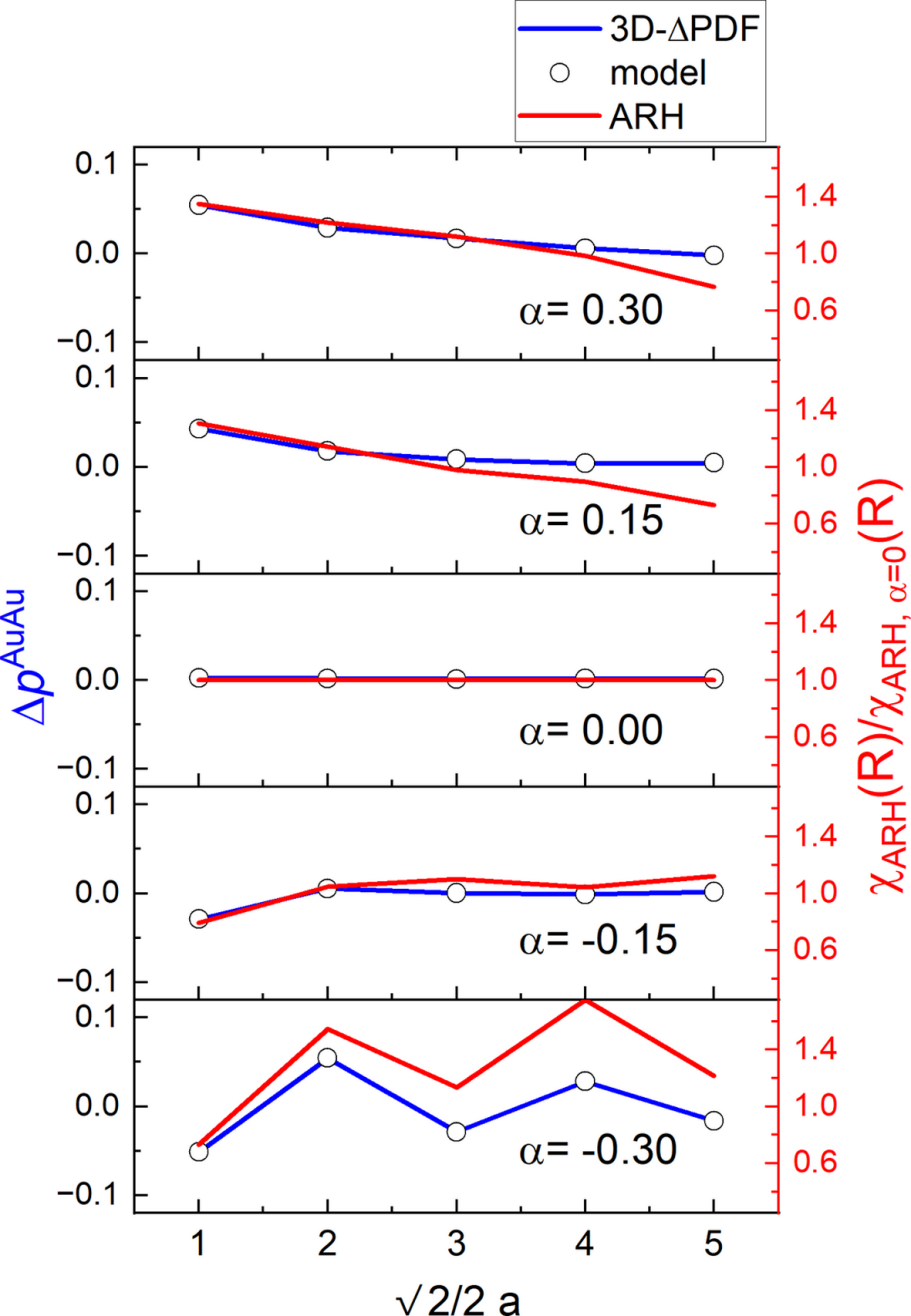
Chemical short-range order parameter analysis along the [110] direction. Δ*p*^AuAu^ fitted from the 3D-ΔPDF (blue lines) compared with the same parameter derived from the model (ground truth, circles) and the normalized signal intensity from the ARH reconstructions (red lines, scale on the right-hand side).

In previous studies, both DS analysis via the 3D-ΔPDF and ARH have been used to investigate variations in local coordination environments. Here, we quantitatively compare the Warren–Cowley short-range order parameter 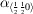 as derived from the model structure, 3D-ΔPDF analysis and ARH. The probability difference 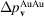, which represents the deviation in the likelihood of finding an Au–Au pair at an interatomic vector **v** in a short-range ordered structure versus a random one, can be derived directly from the model structure (Fig. 3 ▸, and Table S1 in the supporting information) by counting the number of pair occurrences. This parameter can also be refined directly in a 3D-ΔPDF analysis using *Yell* (Fig. 3 ▸, and Table S4 in the supporting information). For the Warren–Cowley short-range order parameter, it holds that 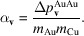
A similar procedure for fitting short-range order parameters from ARH data is not yet well established, and we build our analysis here on the procedure described in Section 2.5. Since ARH signals can be regarded as a measure of the local electron density around a specific element, their intensity also encodes CSRO. By normalizing the integrated intensity as described above, we obtain the red lines in Fig. 3 ▸, oscillating around unity. As seen in the plots of Fig. 3 ▸, their general trend clearly follows that of the 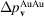 parameters.

The agreement between the 3D-ΔPDF analysis and the ground truth from the structural model is excellent, as expected given the use of noise-free computational data and the specific design of *Yell* (Simonov *et al.*, 2014*b* ▸) for this type of analysis. An analytical expression for the disorder diffuse scattering intensity [see *e.g.* Warren *et al.* (1951 ▸) and Schmidt & Neder (2017 ▸)] directly encodes the parameter α_**v**_ and hence also 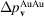 as a linear coefficient. By contrast, the ARH reconstructions show greater deviations – especially for more distant neighbor pairs – than the 3D-ΔPDF, although they still highlight ARH’s potential for quantitative local order investigations.

There are two main sources of error in the ARH technique, both stemming from the relatively small amount of input data (the holograms) in comparison with the output data (large high-resolution 3D volume). These errors are (i) spurious artificial signals that are frequently encountered in holographic methods and (ii) the ‘twin image effect’ (Hayashi *et al.*, 2012 ▸), *i.e.* the mix of real and imaginary parts of the transform algorithm in a centrosymmetric system, which can influence the position and intensity of the actual signals. Both errors are reduced by using several different energies for the holograms.

For both DS and ARH, we expect that the error and uncertainty compared with the ground truth will be much higher for real experimental data. To the best of our knowledge, there is currently no standard procedure to propagate the uncertainty of measured DS data to the experimental 3D-ΔPDF. For this reason, we believe that the statistical errors – derived from the deviations between the model and experimental data and estimated through least-squares refinement as implemented in *Yell* – are typically overestimated. In contrast, systematic errors, arising from data processing routines and atomistic configurations that are not fully captured by the short-range order parameters used in modeling, are generally underestimated. In the experimental case, a reasonable error could be estimated by processing experimental diffuse scattering data on different reconstruction grids, applying various background correction and punch-and-fill procedures, and starting the refinement from several different initial configurations. The average and standard deviation of the refined parameters would probably provide the most reliable uncertainty estimates. However, since we use purely computational data in this study, we expect that the error encountered in the 3D-ΔPDF refinement is sufficiently represented by the uncertainty of the least-squares refinement, with minimal deviation of the refined parameters from the ground truth (Simonov *et al.*, 2014*b* ▸).

In the case of ARH, statistical errors play a major role, because the holograms are experimentally determined from the angular dependence of fluorescence radiation emitted by the sample. This modulation is typically only of the order of about 0.1%, and therefore the accumulation of accurate data is of great importance. On the other hand, intrinsic crystallographic symmetries can be applied to the data to help mitigate this effect. But also in this case, the error propagation from the hologram to the reconstructed data is not well understood or studied.

### 3.2. Size-effect relaxations

**Figure 4:**
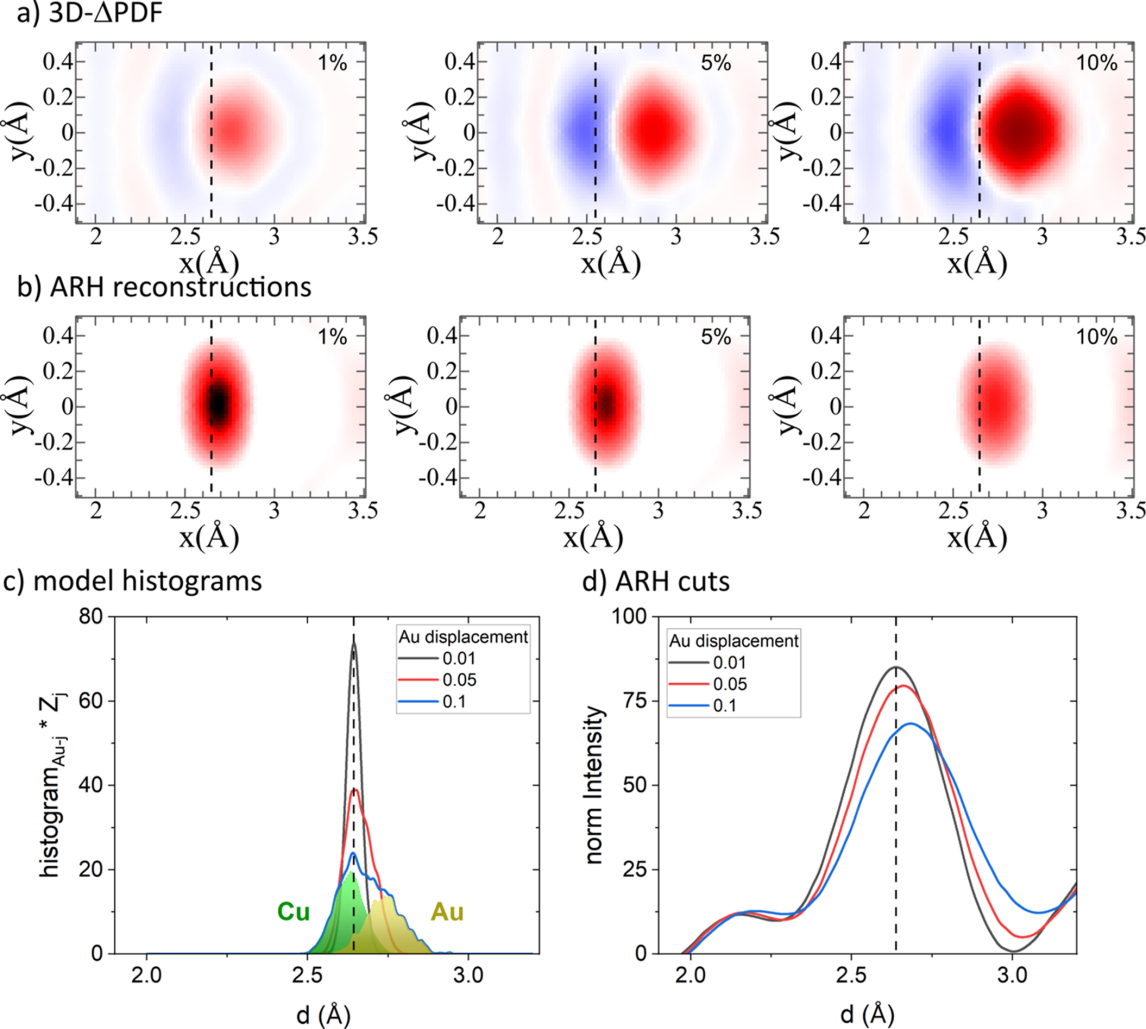
Analysis of the influence of the size effect on the signal shapes. (*a*) 3D-ΔPDFs, (*b*) reconstructions from ARH, (*c*) model histograms (ground truth) and (*d*) slices through the ARH signals shown in panel (*b*).

Substitutional disorder typically leads to local bond-distance relaxations, reflecting the chemical flexibility of the structure. In this context, atoms are statically displaced from their average positions to adjust for variations in their bonding environments. In DS, these relaxations create distinct signatures, manifesting as alternating minima and maxima in the 3D-ΔPDF [Fig. 4 ▸(*a*)].

These signatures can be fitted and interpreted directly using short-range order (SRO) parameters. The (an-)isotropic displacement parameters (ADPs) that are typically refined in an average structure refinement describe a Gaussian mean interatomic vector distribution, **v**, with a width of σ_ADP_. The position of this Gaussian distribution is fixed by the average structure, with the width in the average structure Patterson function given by the sum of the ADPs of the atom at the origin and at the end of the interatomic vector **v**. In the real structure, we assume three types of distinct interatomic vector distributions, **v**_AuAu_, **v**_AuCu_ and **v**_CuCu_. These distributions differ in both position and width (σ_AuAu_, σ_AuCu_ and σ_CuCu_) in the Patterson function from the average structure interatomic vector **v**, which has a width of σ_ADP_. The signature observed in the 3D-ΔPDF is thus the sum of these three real-structure interatomic vector distributions, each weighted by the electron densities in the respective pairs, minus the average structure Gaussian distribution.

If the assumption holds that all the mentioned interatomic vector distributions are accurately represented by Gaussian distributions, the resulting intensity distribution in the 3D-ΔPDF can be fully described by the parameters **v**_AuAu_, **v**_AuCu_, **v**_CuCu_, σ_AuAu_, σ_AuCu_ and σ_CuCu_. This allows for a straightforward quantification of the magnitude and direction of the bond-distance relaxations. However, this assumption may break down for cases involving next-nearest-neighbor configurations, displacement configurations with large displace­ments or displacement configurations with discrete displacements. These limitations affect the applicability of the SRO parameters derived from the explicit fitting of the intensity distribution in the 3D-ΔPDF without a structural model, as implemented in the program *Yell*.

In the ARH atomic image reconstructions, the intensity maxima appear shifted from the expected interatomic vectors of the average structure [Figs. 4 ▸(*b*) and 4 ▸(*d*)]. Unlike 3D-ΔPDF analysis, which isolates deviations from the average structure, ARH directly probes the Patterson function around a specific element. As a result, the average structure inter­atomic vector **v** retains a residual signature in the reconstruc­tion, which in turn leads to the observation that the maxima in the ARH reconstruction appear less displaced from the average structure interatomic vector than those in the 3D-ΔPDF.

Although ARH is not typically employed to derive bond-distance relaxations, fitting the position of the intensity maxima in Fig. 4 ▸(*d*) yields the shift parameters listed in Table 2 ▸. These parameters show reasonable agreement with those obtained from the 3D-ΔPDF analysis, confirming that ARH is indeed a suitable method for investigating local bond distortions.

As with the chemical disorder analysis, the agreement between the model ground truth and the 3D-ΔPDF results is better than that for ARH. In the case of the latter, the spatial resolution is largely limited by the energy range used in the experiment (which is typically close to the X-ray*K* or *L* absorption edge, *i.e.* 5–30 keV), leading to a usual resolution of the order of 0.1 Å. In addition, the accuracy of ARH practically depends on the number of measured holograms; here we used a number of holograms that is comparable to what can be obtained in a typical experiment. For the 3D-ΔPDF, good agreement with the model is expected, since the signatures of the interatomic vector distributions shown in Fig. 4 ▸(*c*) can be accurately represented by Gaussian distributions. However, the generally larger *R* values in the 3D-ΔPDF refinement of the size-effect relaxation, compared with the chemical short-range order (see the supporting information for explicit values), demonstrate two key points: (i) the Gaussian assumption breaks down for further neighbor pairs and (ii) a description based on a limited number of displacement parameter pairs may be insufficient to give a full description of the complexity of structural relaxations in a real-world scenario.

### 3.3. Combination of chemical short-range order and size-effect relaxations

**Figure 5:**
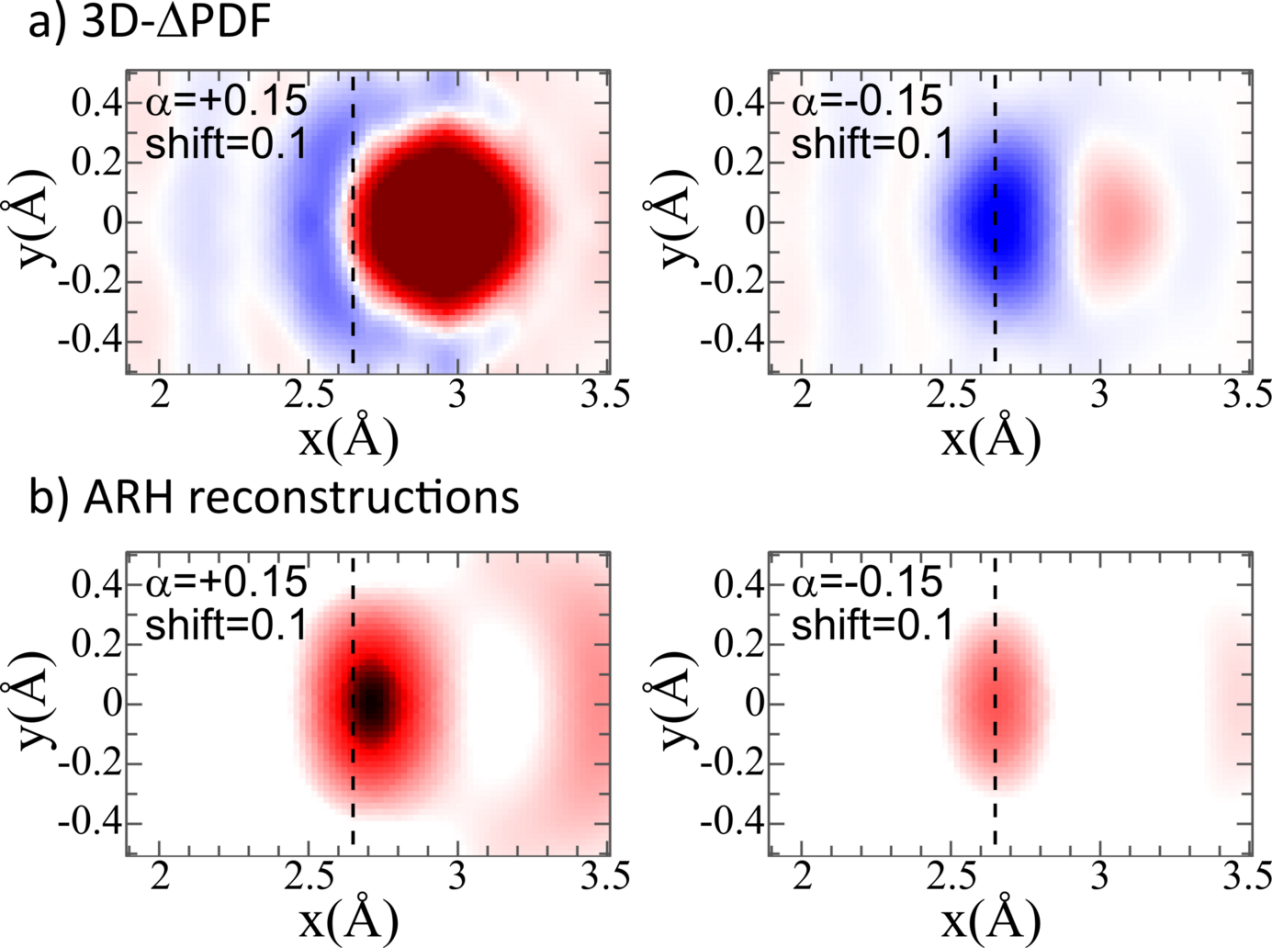
Analysis of the influence of the combined impact of both chemical short-range order and size effect on the signal shape of the first nearest neighbor (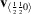). (*a*) 3D-ΔPDFs and (*b*) reconstructions from ARH. The dashed line indicates the position of the nearest neighbor in the average structure.

Analyzing the combined effects of CSRO and size-effect relaxations represents arguably a more realistic scenario for real-world materials than the isolated disorder types discussed in the previous sections. Here, we chose to examine the combination of the largest displacement (Size0.1) with a moderate CSRO of α = ±0.15 (CSRO+0.15 or CSRO−0.15, respectively). The 3D-ΔPDF maps and the reconstructions from ARH are shown in Fig. 5 ▸, and derived first-neighbor Au–Au distances are listed in Table 2 ▸.

In the 3D-ΔPDFs [Fig. 5 ▸(*a*)], the combined effects of CSRO and size relaxations are reflected in both the signal form and its amplitude. The CSRO component tends to dominate the overall appearance; for positive CSRO (α = +0.15), the characteristic maximum signal associated with Au–Au correlations is dominant. Conversely, for negative CSRO (α = −0.15), the minimum signal associated with the absence of expected average pairs is dominant. The more subtle deviations arising specifically from the size-effect relaxations in this combined scenario can be challenging to distinguish and are more easily suppressed by the typical noise and ripples expected in experimental patterns compared with the dominant CSRO signal. While fits to the 3D-ΔPDF effectively capture the degree of CSRO with good precision (as indicated by the fitting parameters in the supporting information), they show a tendency to under/overestimate the specific Au–Au bond distance in these combined cases (Table 2 ▸).

In the ARH atomic image reconstructions [Fig. 5 ▸(*b*)], the CSRO directly impacts the intensity of the signal at specific interatomic vector positions. A stronger signal is observed in the case with more Au–Au pairs (α = +0.15) compared with the case with fewer Au–Au pairs (α = −0.15), reflecting the higher probability of finding a neighboring Au atom around a central Au atom. The complete sets of fitting parameters for the ARH analysis are listed in the supporting information. Importantly, in ARH the CSRO also influences how the size-effect relaxations are observed in the reconstructed data. This is because the shift in the nearest-neighbor signal position is related to the fraction of specific atomic pairs present in the model structure. Therefore, for the case of negative CSRO, where there are fewer Au–Au neighbors, the observed signal is located closer to the average structure position (indicated by the dashed lines in Fig. 5 ▸) than in the case of positive CSRO.

From the 3D-ΔPDF, we refine α = −0.149 for Combined−0.15 and α = 0.234 for Combined+0.15, while we estimate α ≃ −0.321 for Combined−0.15 and α ≃ 0.221 from the ARH reconstruction using equation (5). For both techniques, the estimates are in reasonable agreement with the α_Model_ = −0.157 and α_Model_ = 0.219 that we derive from the pair occurrences in the model structures Combined−0.15 and Combined+0.15, respectively. Similar deviations from the case of pure chemical SRO (see Section 3.1) are observed for both techniques.

Table 2 ▸ provides a quantitative comparison of the Au–Au bond distances derived from the 3D-ΔPDF and ARH for the combined disorder cases, alongside the model ground truth. The 3D-ΔPDF results show a good agreement with the model ground truth for the Au–Au distance, as the deviations are smaller than the variance of the calculated bond distance from the model crystal. In ARH, the deviations are slightly larger, especially for the negative SRO case, where ARH significantly overestimates the bond-distance relaxation. We attribute this misfit to the influence of the Cu–Au correlations that are also present in the ARH reconstruction: in the case of negative short-range order the Cu–Au pairs dominate the first-neighbor pairs and hence also dominate the interatomic distance of first-neighbor pairs around Au atoms. Our fitting procedure assumes that the Cu–Au distance is centered on the average structure interatomic distance and only the Au–Au pairs contribute to the deviation.

A notable qualitative difference between the two methods lies in their overall response to the degree of disorder. In the 3D-ΔPDF, the signal amplitude generally increases with the magnitude of the disorder – specifically, the deviation from the average structure. This is evident as stronger features for cases with larger absolute values of α (supporting information Section S8) or larger size-effect relaxations [Fig. 4 ▸(*a*)]. In contrast, the situation in ARH is more complex: displacement disorder tends to reduce the maximum signal and shift its position, while chemical disorder either increases or decreases the overall signal intensity around a certain interatomic vector, depending on the type of correlation. This fundamental difference arises because the 3D-ΔPDF maps the difference of the real structure from the average structure, which amplifies stronger deviations, whereas ARH reconstructs the local environment directly, where disorder can lead to a smearing out or redistribution of reconstructed intensity.

As noted, resolving the distinct contribution of size-effect relaxations in both the 3D-ΔPDF and ARH can be challenging in the presence of a dominant CSRO signal and experimental noise. We demonstrate that both methods are, in principle, able to capture both effects qualitatively, although it seems that the 3D-ΔPDF is better suited to refining subtle changes in bond-distance relaxations in a binary scenario similar to what we have demonstrated here.

## 4. Concluding remarks and future perspectives

This computational model study successfully demonstrates the applicability and quantitative potential of both DS analysis via the 3D-ΔPDF and ARH for investigating disorder in three-dimensional crystalline systems. Using a binary model mater­ial, we have provided a thorough quantitative comparison of these techniques for characterizing chemical short-range order, size-effect relaxations and their combination, illustrating for the first time that three-dimensional real-space reconstructions from ARH can yield quantitative information about short-range order parameters and identify disorder-driven bond-distance relaxations.

Our results highlight that the 3D-ΔPDF and ARH emphasize different aspects of correlated short-range order and possess distinct strengths and limitations. For simple binary systems like the one we investigated computationally here, the 3D-ΔPDF approach, particularly when combined with dedicated fitting programs like *Yell*, proves highly effective for quantitative analysis of CSRO parameters. We have shown excellent agreement between 3D-ΔPDF-derived CSRO parameters and the model ground truth (Fig. 3 ▸). Similarly, for isolated size-effect relaxations, the 3D-ΔPDF has successfully captured the local bond distortions and derived bond distances, showing good agreement with the model (Table 2 ▸). The method’s strength lies in directly isolating deviations from the average structure and its capacity for quantitative fitting when disorder features can be adequately described by analytical models (*e.g.* Gaussian distributions for relaxations). However, we have noted that this assumption can break down for more complex distortions or more distant neighbors, which would probably require a more complex description in terms of order parameters. The refinements that we show here were performed on noise-free computational data, and therefore we expect that the achieved agreement in terms of refined parameters compared with the ground truth from the model crystal can serve as a benchmark for the best possible agreement. In the real case, it is likely that the refinements would have to be constrained even further. In this study, it was possible, for example, to refine the average structure ADPs during the 3D-ΔPDF refinement. However, in a real experimental scenario, these parameters should typically be fixed to the values obtained from an average structure refinement to avoid strong correlations between refined variables.

ARH, conversely, reconstructs the local atomic environment around a specific atomic species. We have demonstrated that the normalized ARH intensity follows the general trend of CSRO (Fig. 3 ▸) and can be used for a good semi-quantitative estimation of the short-range order parameter α. Peak shifts in ARH reconstructions indicate bond relaxations (Fig. 4 ▸); a quantitative derivation of parameters that describe the relaxation is not standard and proved less accurate than the 3D-ΔPDF, even in the simple binary case investigated here (Table 2 ▸). The ARH results can be regarded as a good semi-quantitative estimate for the bond-distance relaxation. These quantitative differences in our study probably stem from the inherent limitations of ARH related to the spatial resolution, which is determined by the energy range (typically ∼0.1 Å), and the amount of input data (number of holograms), as well as the complexity in interpreting the ARH signal which represents a superposition of scattering contributions from the local environment. The short-range order models were refined against DS data in the 3D-ΔPDF, while for ARH the estimated quantities were extracted from the reconstructed 3D space – also explaining why the agreement between the model ground truth and the simulated data is better for the 3D-ΔPDF analysis than for the ARH reconstructions.

Despite the 3D-ΔPDF showing better quantitative accuracy in this simple binary model study, the situation becomes significantly more nuanced for complex systems involving more than two disordered components. Here, ARH offers a unique and crucial advantage due to its inherent chemical sensitivity, which allows for element-specific local structure determination. This is often diminished or absent in standard DS experiments (*e.g.*X-ray 3D-ΔPDF), where the signal is the sum of all possible correlations, potentially leading to ambiguous results or masking important disorder when contributions average out. For instance, in a ternary alloy, ARH could distinguish element-specific correlations (*e.g.* Au–Ag, Au–Cu) that a single 3D-ΔPDF might not resolve. While combining DS data from different radiation types (X-ray, electron, neutron) can sometimes mitigate this limitation, it introduces significant practical challenges. A case where this could be of practical importance is that of cubic stabilized zirconia (*e.g.* Zr_1−δ_Y_δ_O_2−δ/2_) (Schmidt *et al.*, 2023 ▸). This material adopts the average fluorite structure, and using the 3D-ΔPDF it is impossible to disentangle directly the metal–oxygen correlations around the 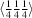 interatomic vector. ARH could yield valuable complementary information and hence, in combination with the 3D-ΔPDF, be used to build a comprehensive, quantitative and reliable disorder model.

Given these complementary strengths, we strongly advocate for a combined approach that exploits the quantitative rigor of 3D-ΔPDF analysis for pair correlations (where applicable and interpretable) alongside the chemical specificity and local environmental insights from ARH. Such a hybrid experimental strategy offers a powerful route to unambiguously solve complex disorder problems that neither technique can fully address in isolation. Readers interested in the complete experimental procedures for both techniques are referred to our previously published work (Stellhorn *et al.*, 2024 ▸), which details the sample requirements, preparation methods and data acquisition protocols for DS and ARH. We envision this integrated approach as a practical strategy to extend the scope of quantitative disorder analysis to increasingly intricate systems, including controversially discussed materials like high-entropy alloys, multivariate metal–organic frameworks (MOFs) and other functional materials where local disorder dictates properties.

## Supplementary Material

Additional details, figures and tables. DOI: 10.1107/S1600576725005977/jur5003sup1.pdf

## Figures and Tables

**Table 1:** Structure identifiers for the ten disordered structures considered in this study with different degrees of chemical short-range order and relaxation of atomic positions The mentioned quantities are employed as targets in the respective Monte Carlo simulations.

Identifier	CSRO	Relaxation
CSRO0	Random	No
CSRO+0.3	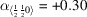	No
CSRO+0.15	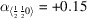	No
CSRO−0.15	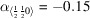	No
CSRO−0.3	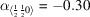	No
Size0.01	Random	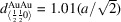
Size0.05	Random	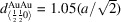
Size0.10	Random	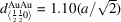
Combined+0.15	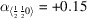	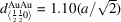
Combined−0.15	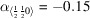	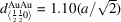

**Table 2:** Au–Au bond distances derived from 3D-ΔPDF analysis and ARH compared with the ground truth calculated by averaging all the first-neighbor Au–Au bond distances in the model crystal

Identifier	3D-ΔPDF	ARH	Model
Size0.01	2.65 Å	2.61 Å	2.66 (2) Å
Size0.05	2.69 Å	2.70 Å	2.70 (3) Å
Size0.10	2.74 Å	2.82 Å	2.75 (6) Å
Combined+0.15	2.77 Å	2.72 Å	2.76 (8) Å
Combined−0.15	2.71 Å	2.86 Å	2.74 (4) Å
