# Supplementary material for: Solving disorder in (3D) real space: a comparative study of the three-dimensional difference pair distribution function and atomic resolution holography reconstructions
Source: J Appl Crystallogr. 2025 Aug 8;58(Pt 5):1605–14. doi: 10.1107/S1600576725005977 (PMC12502872; doi:10.1107/S1600576725005977)
Supplement: Supplementary file 1 [file j-58-01605-sup1.pdf]

JOURNAL OF APPLIED CRYSTALLOGRAPHY

Volume 58 (2025)

Supporting information for article:

Solving disorder in (3D) real space – a comparative study of the three-dimensional difference pair distribution function (3D-ΔPDF) and atomic resolution holography reconstructions

Jens R. Stellhorn, Arianna Minelli, Emily G. Meekel and Ella M. Schmidt

Contents

1 Parameters for the Monte Carlo Simulation S3

2 Pair-probabilities from the structural models S4

3 Relaxations from the structural models S6

4 Yell refinement parameters for chemical short range order S7

5 Yell refinement parameters for distance relaxation S9

6 Yell refinement parameters for combined refinements S11

7 ARH refinement parameters for Gaussian fits S14

8 Sections of the simulated 3D-ΔPDFs and ARH reconstructions S15

1 Parameters for the Monte Carlo Simulation

To generate the disordered structures a forward Monte Carlo simulation as implemented in the DISCUS program was used. For each of the 4 symmetry equivalent sites in the unit-cell the 12 nearest neighbor vectors were grouped into neighborhood 1, to which target 1 applies. `n[1]` specifies the number of atoms in the crystal structure, here 4 000.

The following parameters were used for chemical short range order:

```
set target, 1, corr, Au, Cu, +0.15, 0.0, CORR
set temp, 0.1
set mode, 1.0, swchem, all
set cyc, 200*n[1]
set feed, 10*n[1]
```

The following parameters were used for size-effect relaxations:

```
set target, 1, spring, Au, Au, 0.5*sqrt(2)*lat[1]*(1+daa), 1000.
set target, 1, spring, Au, Cu, 0.5*sqrt(2)*lat[1]*(1+dab), 1000.
set target, 1, spring, Cu, Cu, 0.5*sqrt(2)*lat[1]*(1+dbb), 1000.
set temp, 0.1
set mode,0.0, swchem
set mode, 1.0, shift, all
set move,Cu, 0.02,0.02,0.02
set move,Au, 0.02,0.02,0.02
set cyc, 200*n[1]
set feed, 10*n[1]
```

`daa` corresponds to δ of the main text, `dab` is set to 0 and `dbb` is set to $-\delta/9$. The spring potentials are hook-potentials, where the last parameter in each line defines the depth of the potential.

2 Pair-probabilities from the structural models

The generated structural models were analysed for their respective pair-probabilities. The probability to find an Au-Au pair at a certain interatomic vector $\vec{v}$ are listed in Table S1.

Table S1: Probability to find a Au-Au pair separated by a vector $\vec{v}$ for the structural models.

$\vec{v}$	**CSRO0**	**CSRO+0.3**	**CSRO+0.15**	**CSRO-0.15**	**CSRO-0.3**
$\langle\frac{1}{2}\ \frac{1}{2}\ 0\rangle$	0.0703	0.1163	0.1051	0.0312	0.0126
$\langle 1\ 0\ 0\rangle$	0.0630	0.0992	0.0869	0.0862	0.1672
$\langle 1\ 1\ 0\rangle$	0.0655	0.0921	0.0794	0.0663	0.1288
$\langle\frac{3}{2}\ \frac{1}{2}\ 0\rangle$	0.0627	0.0863	0.0745	0.0531	0.0252
$\langle 1\ \frac{1}{2}\ \frac{1}{2}\rangle$	0.0635	0.0960	0.0823	0.0654	0.0547
$\langle 1\ 1\ 1\rangle$	0.0622	0.0854	0.0746	0.0616	0.1014
$\langle\frac{3}{2}\ 1\frac{1}{2}\rangle$	0.0629	0.0831	0.0716	0.0593	0.0514
$\langle\frac{3}{2}\ \frac{3}{2}\ 0\rangle$	0.0629	0.0813	0.0689	0.0597	0.0340
$\langle 1\frac{3}{2}\ \frac{3}{2}\rangle$	0.0633	0.0772	0.0677	0.0594	0.0530
$\langle 2\ 0\ 0\rangle$	0.0605	0.0793	0.0699	0.0643	0.1370
$\langle 2\ 1\ 0\rangle$	0.0629	0.0775	0.0678	0.0603	0.1133
$\langle 2\ 2\ 0\rangle$	0.0651	0.0716	0.0640	0.0592	0.1049
$\langle 2\ \frac{1}{2}\ \frac{1}{2}\rangle$	0.0645	0.0783	0.0680	0.0648	0.0624
$\langle 2\ \frac{3}{2}\ \frac{1}{2}\rangle$	0.0628	0.0752	0.0663	0.0613	0.0580
$\langle 2\ 1\ 1\rangle$	0.0639	0.0758	0.0670	0.0597	0.0946
$\langle 2\ 2\ 1\rangle$	0.0635	0.0713	0.0658	0.0592	0.0910
$\langle\frac{5}{2}\ \frac{1}{2}\ 0\rangle$	0.0633	0.0732	0.0659	0.0584	0.0341
$\langle\frac{5}{2}\ \frac{3}{2}\ 0\rangle$	0.0656	0.0705	0.0634	0.0613	0.0402
$\langle\frac{5}{2}\ \frac{5}{2}\ 0\rangle$	0.0630	0.0662	0.0639	0.0618	0.0452
$\langle 3\ 0\ 0\rangle$	0.0667	0.0691	0.0643	0.0621	0.1167
$\langle 3\ 1\ 0\rangle$	0.0636	0.0666	0.0640	0.0605	0.1015
$\langle 3\ 2\ 0\rangle$	0.0636	0.0657	0.0628	0.0579	0.0954
$\langle\frac{5}{2}\ 1\ \frac{1}{2}\rangle$	0.0646	0.0721	0.0649	0.0599	0.0515

$\langle \frac{5}{2}\ 2\ \frac{1}{2} \rangle$	0.0639	0.0689	0.0638	0.0601	0.0559
$\langle 3\ \frac{1}{2}\ \frac{1}{2} \rangle$	0.0642	0.0685	0.0652	0.0606	0.0671
$\langle 3\ \frac{3}{2}\ \frac{1}{2} \rangle$	0.0633	0.0659	0.0636	0.0599	0.0635
$\langle \frac{5}{2}\ \frac{3}{2}\ 1 \rangle$	0.0647	0.0694	0.0645	0.0596	0.0540
$\langle 3\ 1\ 1 \rangle$	0.0646	0.0668	0.0632	0.0611	0.0883
$\langle 3\ 2\ 1 \rangle$	0.0648	0.0635	0.0639	0.0606	0.0848
$\langle \frac{5}{2}\ \frac{5}{2}\ 1 \rangle$	0.0661	0.0642	0.0637	0.0607	0.0554
$\langle 2\ \frac{3}{2}\ \frac{3}{2} \rangle$	0.0604	0.0707	0.0662	0.0620	0.0570
$\langle 3\ \frac{3}{2}\ \frac{3}{2} \rangle$	0.0625	0.0636	0.0633	0.0598	0.0631
$\langle \frac{5}{2}\ 2\ \frac{3}{2} \rangle$	0.0632	0.0651	0.0650	0.0603	0.0563
$\langle 3\ \frac{5}{2}\ \frac{3}{2} \rangle$	0.0636	0.0607	0.0637	0.0603	0.0630
$\langle 2\ 2\ 2 \rangle$	0.0620	0.0648	0.0649	0.0586	0.0892
$\langle \frac{5}{2}\ \frac{5}{2}\ 2 \rangle$	0.0641	0.0614	0.0648	0.0607	0.0565
$\langle 3\ 2\ 2 \rangle$	0.0641	0.0611	0.0638	0.0600	0.0829

3 Relaxations from the structural models

The Au-Au bond distance histograms that are shown in Figure 4(c) of the main text were fitted using one Gaussian distribution to quantify the achieved Au-Au distance relaxation. The resulting parameters are listed in Table S2.

Table S2: Gaussian fits to the Au-Au first neighbour histogram compared to average bond distance with standard deviation.

Identifier	Relative relaxation (r.l.u.) Gaussian fit	FWHM(Å) Gaussian fit	Relative relaxation (r.l.u.) Average value
Size0.01	$2.04(16) \cdot 10^{-3}$	$5.18(8) \cdot 10^{-2}$	$(3.92 \pm 8.32) \cdot 10^{-3}$
Size0.05	$1.74(3) \cdot 10^{-2}$	$8.40(11) \cdot 10^{-2}$	$(1.92 \pm 1.36) \cdot 10^{-2}$
Size0.1	$3.58(4) \cdot 10^{-2}$	$1.4(2) \cdot 10^{-1}$	$(3.81 \pm 2.34) \cdot 10^{-2}$
Combined+0.15	$4.16(5) \cdot 10^{-3}$	$7.2(2) \cdot 10^{-2}$	$(4.21 \pm 1.63) \cdot 10^{-2}$
Combined-0.15	$3.49(4) \cdot 10^{-3}$	$9.8(2) \cdot 10^{-2}$	$(3.70 \pm 2.84) \cdot 10^{-2}$

Furthermore, the relaxation of further neighbors was calculated by averaging all occurring Au-Au bond distances in the model. These are listed in Table S3

Table S3: Size effect relaxation for Au-Au pairs for different interatomic vectors $\vec{v}$ in r.l.u. as calculated by averaging all respective Au-Au bond distances from the model structure.

$\vec{v}$	Size0.01	Size0.05	Size0.10	Combined+0.15	Combined-0.15
$\langle\frac{1}{2}\ \frac{1}{2}\ 0\rangle$	0.003921	0.019217	0.038144	0.042096	0.036985
$\langle 1\ 0\ 0\rangle$	0.000115	0.001060	0.001471	0.019968	-0.005862
$\langle 1\ 1\ 0\rangle$	0.000334	0.001519	0.003066	0.015926	-0.000908
$\langle 2\ 0\ 0\rangle$	-0.000003	0.000440	0.000459	0.006129	-0.001135
$\langle\frac{3}{2}\ \frac{1}{2}\ 0\rangle$	0.000055	0.000393	0.000611	0.010022	0.002177
$\langle 1\ \frac{1}{2}\ \frac{1}{2}\rangle$	0.000305	0.001433	0.003373	0.018254	-0.001708

4 Yell refinement parameters for chemical short range order

The Yell refinement of the generated diffuse scattering patterns yielded the respective pair correlations listed in Table S4, the achieved R-vaules are listed in Table S5.

Table S4: Probability to find a Au-Au pair separated by a vector $\vec{v}$ from the Yell refinement.

$\vec{v}$	CSRO0	CSRO+0.3	CSRO+0.15	CSRO-0.15	CSRO-0.3
$\langle\frac{1}{2}\ \frac{1}{2}\ 0\rangle$	0.066799(1)	0.117515(2)	0.106813(2)	0.031327(1)	0.013229(1)
$\langle 1\ 0\ 0\rangle$	0.067738(2)	0.100416(1)	0.089112(2)	0.085293(1)	0.160573(1)
$\langle 1\ 1\ 0\rangle$	0.066522(1)	0.091763(2)	0.081664(2)	0.065658(1)	0.119102(1)
$\langle\frac{3}{2}\ \frac{1}{2}\ 0\rangle$	0.065993(1)	0.085963(2)	0.076917(1)	0.053198(1)	0.026640(1)
$\langle 1\ \frac{1}{2}\ \frac{1}{2}\rangle$	0.066294(1)	0.095498(2)	0.084701(1)	0.065914(1)	0.059035(0)
$\langle 1\ 1\ 1\rangle$	0.062890(2)	0.084232(4)	0.077573(3)	0.060881(2)	0.089563(1)
$\langle\frac{3}{2}\ 1\frac{1}{2}\rangle$	0.065711(1)	0.081781(2)	0.075011(1)	0.059239(1)	0.055314(0)
$\langle\frac{3}{2}\ \frac{3}{2}\ 0\rangle$	0.066186(2)	0.079748(3)	0.072384(2)	0.060539(1)	0.035868(1)
$\langle 1\frac{3}{2}\ \frac{3}{2}\rangle$	0.067309(2)	0.075098(3)	0.071350(2)	0.059965(1)	0.056891(1)
$\langle 2\ 0\ 0\rangle$	0.070334(2)	0.079208(1)	0.072502(3)	0.063653(2)	0.128088(1)
$\langle 2\ 1\ 0\rangle$	0.066615(1)	0.075884(2)	0.070762(2)	0.059950(1)	0.102445(1)
$\langle 2\ 2\ 0\rangle$	0.068040(3)	0.068648(4)	0.067743(3)	0.059096(2)	0.092832(1)
$\langle 2\ \frac{1}{2}\ \frac{1}{2}\rangle$	0.066859(1)	0.077078(2)	0.071046(2)	0.065574(1)	0.067826(1)
$\langle 2\ \frac{3}{2}\ \frac{1}{2}\rangle$	0.067685(1)	0.072478(2)	0.070002(2)	0.061044(1)	0.063037(1)
$\langle 2\ 1\ 1\rangle$	0.066739(2)	0.073088(3)	0.070287(2)	0.059542(1)	0.082156(1)
$\langle 2\ 2\ 1\rangle$	0.066863(2)	0.066997(4)	0.070188(3)	0.059205(2)	0.078226(1)
$\langle\frac{5}{2}\ \frac{1}{2}\ 0\rangle$	0.066925(1)	0.071601(2)	0.068910(2)	0.057989(1)	0.036130(1)
$\langle\frac{5}{2}\ \frac{3}{2}\ 0\rangle$	0.067857(2)	0.067369(3)	0.066921(2)	0.062311(1)	0.042483(1)
$\langle\frac{5}{2}\ \frac{5}{2}\ 0\rangle$	0.069305(4)	0.060589(6)	0.068506(5)	0.061965(3)	0.048251(2)
$\langle 3\ 0\ 0\rangle$	0.067731(3)	0.067895(1)	0.068342(4)	0.061667(3)	0.105408(2)
$\langle 3\ 1\ 0\rangle$	0.069033(2)	0.063283(3)	0.067997(3)	0.060227(2)	0.089171(1)
$\langle 3\ 2\ 0\rangle$	0.069852(3)	0.059901(4)	0.067393(3)	0.058624(2)	0.082643(1)
$\langle\frac{5}{2}\ 1\ \frac{1}{2}\rangle$	0.066442(1)	0.069375(2)	0.068410(2)	0.059568(1)	0.055260(1)
$\langle\frac{5}{2}\ 2\ \frac{1}{2}\rangle$	0.068996(1)	0.063672(3)	0.068334(2)	0.059620(1)	0.060554(1)

$\langle 3\ \frac{1}{2}\ \frac{1}{2}\rangle$	0.067041(2)	0.065864(3)	0.069735(2)	0.062019(2)	0.073455(1)
$\langle 3\ \frac{3}{2}\ \frac{1}{2}\rangle$	0.068258(2)	0.060290(3)	0.068350(2)	0.060041(1)	0.069171(1)
$\langle \frac{5}{2}\frac{3}{2}\ 1\rangle$	0.068020(1)	0.064978(3)	0.068679(2)	0.060322(1)	0.057632(1)
$\langle 3\ 1\ 1\rangle$	0.068324(2)	0.061911(4)	0.067802(3)	0.059969(2)	0.075178(1)
$\langle 3\ 2\ 1\rangle$	0.068753(2)	0.055749(4)	0.070089(3)	0.059445(2)	0.071794(1)
$\langle \frac{5}{2}\ \frac{5}{2}\ 1\rangle$	0.071695(3)	0.056417(5)	0.070216(4)	0.061407(3)	0.059128(2)
$\langle 2\ \frac{3}{2}\ \frac{3}{2}\rangle$	0.068017(2)	0.066682(3)	0.070604(3)	0.061370(2)	0.061510(1)
$\langle 3\ \frac{3}{2}\ \frac{3}{2}\rangle$	0.070315(3)	0.056341(5)	0.069422(4)	0.060357(3)	0.068199(2)
$\langle \frac{5}{2}\ 2\ \frac{3}{2}\rangle$	0.069957(2)	0.058463(3)	0.070913(3)	0.060979(1)	0.060614(1)
$\langle 3\ \frac{5}{2}\ \frac{3}{2}\rangle$	0.072605(3)	0.048880(5)	0.073007(4)	0.060161(2)	0.067986(1)
$\langle 2\ 2\ 2\rangle$	0.071595(5)	0.059287(8)	0.071128(6)	0.057231(4)	0.076912(3)
$\langle \frac{5}{2}\ \frac{5}{2}\ 2\rangle$	0.071326(4)	0.051569(7)	0.073760(5)	0.061574(3)	0.060510(2)
$\langle 3\ 2\ 2\rangle$	0.071126(4)	0.048848(7)	0.070952(6)	0.057639(3)	0.070094(2)

Table S5: R-values of Yell refinements of chemical short range order.

Identifier	CSRO0	CSRO+0.3	CSRO+0.15	CSRO-0.15	CSRO-0.3
R-value	0.0476	0.0437	0.0492	0.0333	0.123

5 Yell refinement parameters for distance relaxation

For the yell refinements of the diffuse scattering with distance relaxations several types of parameters were refined:

- Overall ADP parameter (Listed in Table S6)
- Pair-probabilities for the six nearest neighbours (Listed in Table S7)
- Size-effect parameters for the six nearest neighbours (Listed in Table S8)
- ADP correlation parameters for the six nearest neighbours (Listed in Table S9)

Table S6: R-values of and refined ADP of Yell refinements for size effect structures.

Identifier	Size0.01	Size0.05	Size0.10
R-value	0.0427	0.0369	0.0567
ADP (Å^2)	0.0008197(2)	0.0014043(2)	0.0046497(12)

Table S7: Probability to find a Au-Au pair separated by a vector $\vec{v}$ from the Yell refinement for size effect structures.

$\vec{v}$	Size0.01	Size0.05	Size0.10
$\langle \frac{1}{2}\ \frac{1}{2}\ 0\rangle$	0.064147(2)	0.064156(2)	0.064304(4)
$\langle 1\ 0\ 0\rangle$	0.065255(3)	0.065100(3)	0.064595(5)
$\langle 1\ 1\ 0\rangle$	0.062067(3)	0.062041(2)	0.061698(5)
$\langle 2\ 0\ 0\rangle$	0.064480(3)	0.064527(3)	0.064320(8)
$\langle \frac{3}{2}\ \frac{1}{2}\ 0\rangle$	0.062908(2)	0.062895(2)	0.062597(3)
$\langle 1\ \frac{1}{2}\ \frac{1}{2}\rangle$	0.063149(2)	0.063088(2)	0.062608(3)

Table S8: Size effect relaxation for Au-Au pairs in direction of inter-atomic vector $\vec{v}$ in r.l.u. as refined by Yell. Compare to the model values in Table S3.

$\vec{v}$	Size0.01	Size0.05	Size0.10
$\langle\frac{1}{2}\ \frac{1}{2}\ 0\rangle$	0.0035461(10)	0.0185785(15)	0.035039(5)
$\langle 1\ 0\ 0\rangle$	-0.000317(2)	0.002084(2)	0.002200(5)
$\langle 1\ 1\ 0\rangle$	0.0004879(11)	0.0021867(11)	0.003316(3)
$\langle 2\ 0\ 0\rangle$	-0.000269(2)	0.000748(2)	0.000636(6)
$\langle\frac{3}{2}\ \frac{1}{2}\ 0\rangle$	0.0000549(10)	0.0007710(11)	0.000057(2)
$\langle 1\ \frac{1}{2}\ \frac{1}{2}\rangle$	0.0004984(9)	0.0025867(9)	0.004561(2)

Table S9: ADP Pair-correlations for Au-Au pairs at inter-atomic vector $\vec{v}$ in Å^2 as refined by Yell.

$\vec{v}$	component	Size0.01	Size0.05	Size0.10
$\langle\frac{1}{2}\ \frac{1}{2}\ 0\rangle$	xx	0.00061458(17)	0.00060625(17)	0.0019614(7)
$\langle\frac{1}{2}\ \frac{1}{2}\ 0\rangle$	xy	0.00011910(15)	0.00003183(15)	-0.0001325(4)
$\langle\frac{1}{2}\ \frac{1}{2}\ 0\rangle$	zz	0.0005962(2)	0.0008497(2)	0.0031020(9)
$\langle 1\ 0\ 0\rangle$	xx	0.0003506(3)	-0.0000526(3)	-0.0004415(10)
$\langle 1\ 0\ 0\rangle$	yy	0.0004215(2)	0.0004894(2)	0.0020804(8)
$\langle 1\ 1\ 0\rangle$	xx	0.00021395(17)	0.00006806(17)	0.0002044(5)
$\langle 1\ 1\ 0\rangle$	xy	0.00009566(16)	-0.00003544(17)	-0.0001769(5)
$\langle 1\ 1\ 0\rangle$	zz	0.0002118(2)	0.0001912(2)	0.0008559(7)
$\langle 2\ 0\ 0\rangle$	xx	0.0000320(3)	0.0000306(3)	0.0002569(10)
$\langle 2\ 0\ 0\rangle$	yy	0.0000768(2)	0.0000607(2)	0.0003093(7)
$\langle\frac{3}{2}\ \frac{1}{2}\ 0\rangle$	xx	0.00011554(17)	0.00003737(17)	0.0000381(5)
$\langle\frac{3}{2}\ \frac{1}{2}\ 0\rangle$	xy	0.00005015(11)	0.00000264(12)	0.0000358(3)
$\langle\frac{3}{2}\ \frac{1}{2}\ 0\rangle$	yy	0.00015839(17)	0.00013275(17)	0.0006874(5)
$\langle\frac{3}{2}\ \frac{1}{2}\ 0\rangle$	zz	0.00016343(17)	0.00011919(17)	0.0005611(5)
$\langle 1\ \frac{1}{2}\ \frac{1}{2}\rangle$	xx	0.00024268(16)	0.00003440(17)	0.0000487(5)
$\langle 1\ \frac{1}{2}\ \frac{1}{2}\rangle$	yy	0.00025772(12)	0.00022933(12)	0.0009072(4)
$\langle 1\ \frac{1}{2}\ \frac{1}{2}\rangle$	xy	0.00005674(8)	-0.00003098(8)	-0.0001802(2)
$\langle 1\ \frac{1}{2}\ \frac{1}{2}\rangle$	yz	0.00002876(11)	-0.00004286(11)	-0.0002267(3)

6 Yell refinement parameters for combined refinements

For the yell refinements of the diffuse scattering with chemical short range order and distance relaxations several types of parameters were refined:

- Overall ADP parameter (Listed in Table S10)
- Pair-probabilities for the six nearest neighbours (Listed in Table S11)
- Size-effect parameters for the six nearest neighbours (Listed in Table S12)
- ADP correlation parameters for the six nearest neighbours (Listed in Table S13)

Table S10: R-values of and refined ADP of Yell refinements for combined structures.

Identifier	Combined-0.15	Combined+0.15
R-value	0.1292	0.2734
ADP (Å^2)	0.0026251(16)	0.009082(7)

Table S11: Probability to find a Au-Au pair separated by a vector $\vec{v}$ from the Yell refinement for combined structures.

$\vec{v}$	Combined-0.15	Combined+0.15
$\langle \frac{1}{2}\ \frac{1}{2}\ 0\rangle$	0.03630(7)	0.10895(2)
$\langle 1\ 0\ 0\rangle$	0.09008(9)	0.08874(3)
$\langle 1\ 1\ 0\rangle$	0.07017(6)	0.07977(3)
$\langle 2\ 0\ 0\rangle$	0.06798(9)	0.06922(4)
$\langle \frac{3}{2}\ \frac{1}{2}\ 0\rangle$	0.05732(4)	0.07383(2)
$\langle 1\ \frac{1}{2}\ \frac{1}{2}\rangle$	0.07015(4)	0.08214(2)

Table S12: Size effect relaxation for Au-Au pairs in combined structures in direction of inter-atomic vector $\vec{v}$ in r.l.u. as refined by Yell. Compare to the model values in Table S3.

$\vec{v}$	Combined-0.15	Combined+0.15
$\langle \frac{1}{2}\ \frac{1}{2}\ 0\rangle$	0.026717(11)	0.04805(2)
$\langle 1\ 0\ 0\rangle$	-0.015811(8)	0.04794(5)
$\langle 1\ 1\ 0\rangle$	-0.001412(4)	0.00968(3)
$\langle 2\ 0\ 0\rangle$	-0.003445(10)	0.00281(4)
$\langle \frac{3}{2}\ \frac{1}{2}\ 0\rangle$	0.004716(5)	0.00579(2)
$\langle 1\ \frac{1}{2}\ \frac{1}{2}\rangle$	0.002963(4)	0.01134(2)

Table S13: ADP Pair-correlations for Au-Au pairs at inter-atomic vector $\vec{v}$ in Å^2 as refined by Yell for combined structures.

$\vec{v}$	component	Combined-0.15	Combined+0.15
$\langle\frac{1}{2}\ \frac{1}{2}\ 0\rangle$	xx	0.0017103(12)	0.006140(7)
$\langle\frac{1}{2}\ \frac{1}{2}\ 0\rangle$	xy	0.0001426(10)	-0.000680(2)
$\langle\frac{1}{2}\ \frac{1}{2}\ 0\rangle$	zz	0.0023821(16)	0.007858(7)
$\langle 1\ 0\ 0\rangle$	xx	0.0004586(12)	0.001564(9)
$\langle 1\ 0\ 0\rangle$	yy	0.0010436(10)	0.007071(8)
$\langle 1\ 1\ 0\rangle$	xx	0.0006604(8)	0.001679(6)
$\langle 1\ 1\ 0\rangle$	xy	0.0001839(7)	-0.002458(5)
$\langle 1\ 1\ 0\rangle$	zz	0.0005776(11)	0.005041(8)
$\langle 2\ 0\ 0\rangle$	xx	0.0000991(16)	-0.002343(14)
$\langle 2\ 0\ 0\rangle$	yy	0.0003001(11)	0.003035(8)
$\langle\frac{3}{2}\ \frac{1}{2}\ 0\rangle$	xx	0.0005340(8)	-0.001046(6)
$\langle\frac{3}{2}\ \frac{1}{2}\ 0\rangle$	xy	0.0001290(6)	-0.001287(3)
$\langle\frac{3}{2}\ \frac{1}{2}\ 0\rangle$	yy	0.0005786(8)	0.004086(6)
$\langle\frac{3}{2}\ \frac{1}{2}\ 0\rangle$	zz	0.0004562(8)	0.004516(6)
$\langle 1\ \frac{1}{2}\ \frac{1}{2}\rangle$	xx	0.0006515(8)	0.001519(6)
$\langle 1\ \frac{1}{2}\ \frac{1}{2}\rangle$	yy	0.0006911(6)	0.004783(6)
$\langle 1\ \frac{1}{2}\ \frac{1}{2}\rangle$	xy	0.0001665(3)	-0.001396(2)
$\langle 1\ \frac{1}{2}\ \frac{1}{2}\rangle$	yz	-0.0000641(5)	-0.000880(2)

7 ARH refinement parameters for Gaussian fits

The nearest neighbor signals of the ARH reconstructions in real space were fitted with Gaussian functions. As compared to the 3D-Δ PDF refinement, this is a much simpler form of deriving the parameters.

For the distance relaxtion models, the Au-Au distances are derived from the fitted distances assuming that the Au-Cu distance remains at the value given from the average lattice, and a probability of Au-Au pairs from the overall SRO parameter of the system.

Table S14: Fitting parameters for the Gaussian fits to the nearest neighbors in the ARH reconstructed image intensities for the distance relaxtion models.

	Size0.01	Size0.05	Size0.1	Combined-0.15	Combined+0.15
peak position (Å)	2.63	2.66	2.69	2.65	2.72
FWHM (Å)	0.33	0.35	0.40	0.47	0.56
relative Area	1.00	0.98	0.95	0.71	1.20
derived: Au-Au dist (Å)	2.61	2.70	2.82	2.71	2.86

Table S15: Relative areas of the ARH fits for the CSRO models along the $\langle 1\ 1\ 0 \rangle$ direction.

	CSRO-0.3	CSRO-0.15	CSRO0	CSRO+0.15	CSRO+0.3
$\langle \frac{1}{2}\ \frac{1}{2}\ 0 \rangle$	0.730	0.794	1.000	1.308	1.351
$\langle 1\ 1\ 0 \rangle$	1.543	1.046	1.000	1.145	1.217
$\langle \frac{3}{2}\ \frac{3}{2}\ 0 \rangle$	1.134	1.099	1.000	0.980	1.119
$\langle 2\ 2\ 0 \rangle$	1.752	1.045	1.000	0.897	0.984
$\langle \frac{5}{2}\ \frac{5}{2}\ 0 \rangle$	1.214	1.121	1.000	0.733	0.767

8 Sections of the simulated 3D-ΔPDFs and ARH reconstructions

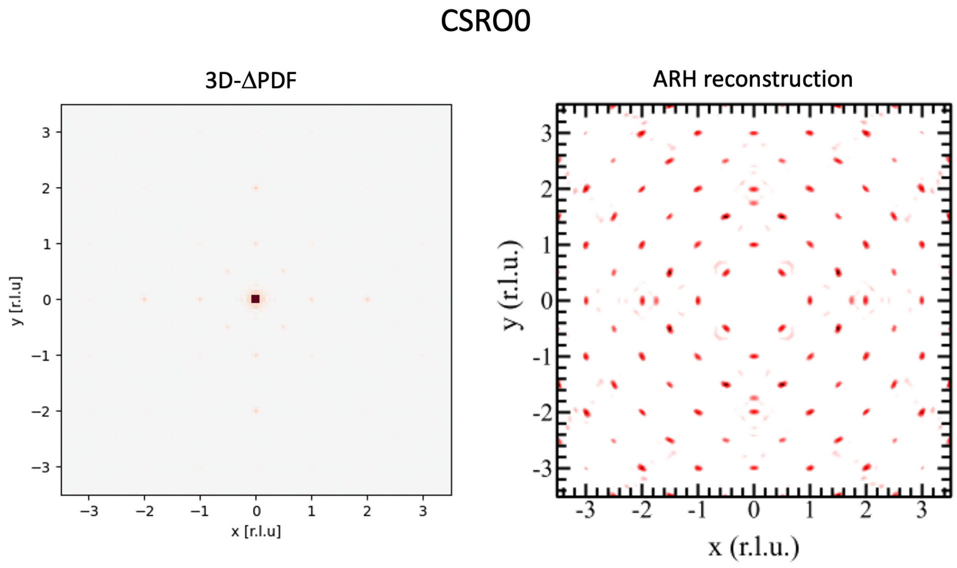

Figure S1: $xy0$-section of the CSRO0 structure for the 3D-ΔPDF (left) and for the ARH reconstruction (right).

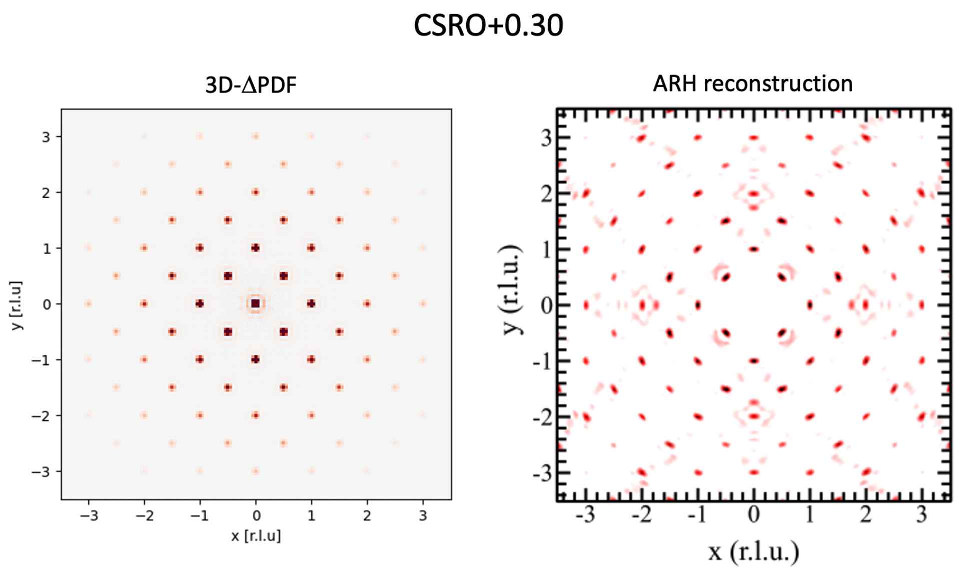

Figure S2: $xy0$-section of the CSRO+0.30 structure for the 3D-ΔPDF (left) and for the ARH reconstruction (right).

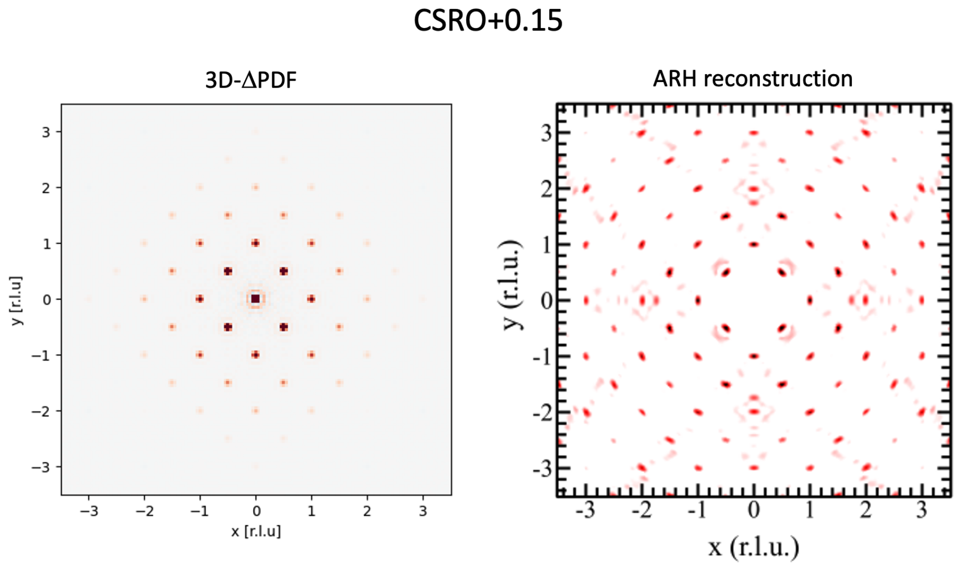

Figure S3: $xy0$-section of the CSRO+0.30 structure for the 3D-ΔPDF (left) and for the ARH reconstruction (right).

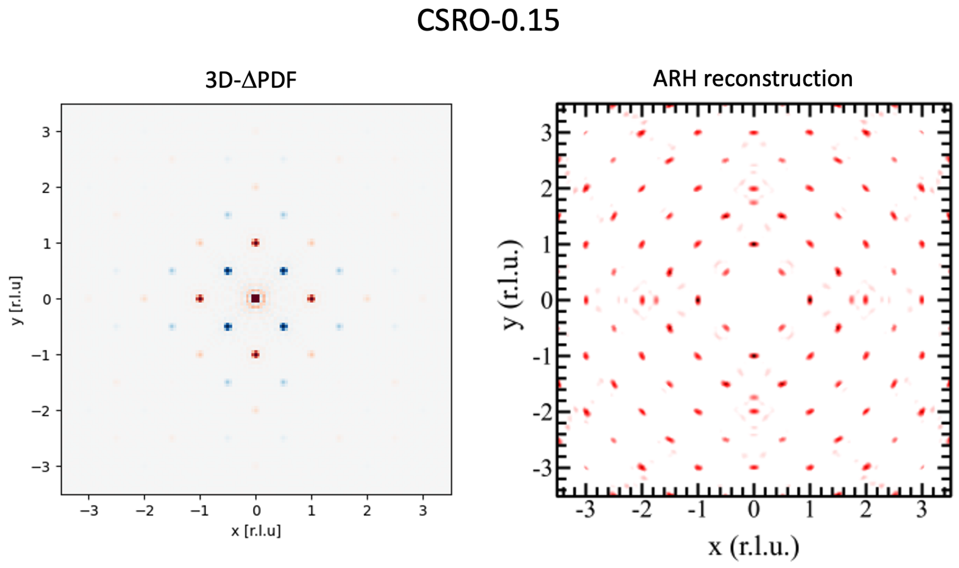

Figure S4: $xy0$-section of the CSRO-0.15 structure for the 3D-ΔPDF (left) and for the ARH reconstruction (right).

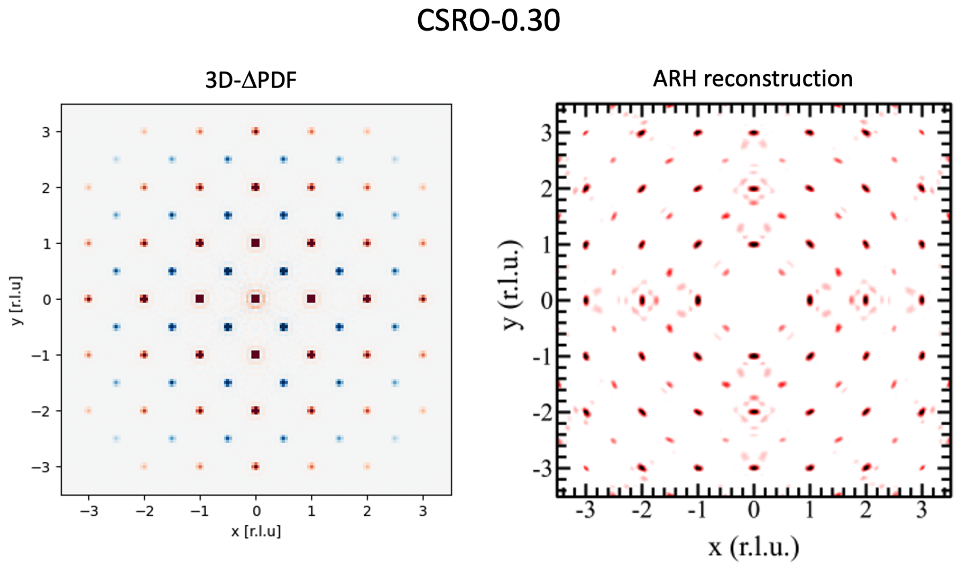

Figure S5: $xy0$-section of the CSRO-0.30 structure for the 3D-ΔPDF (left) and for the ARH reconstruction (right).

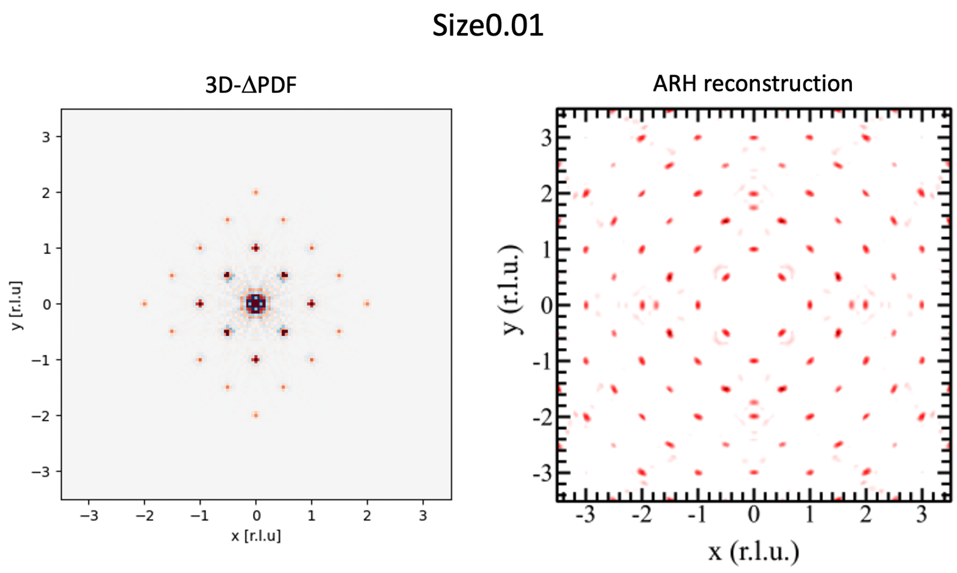

Figure S6: $xy0$-section of the Size0.01 structure for the 3D-ΔPDF (left) and for the ARH reconstruction (right).

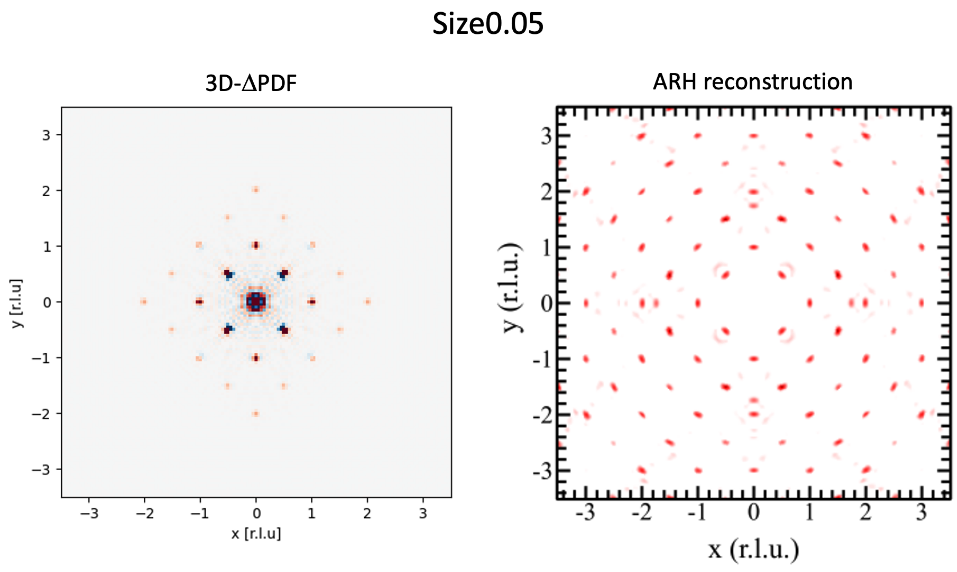

Figure S7: $xy0$-section of the Size0.05 structure for the 3D-ΔPDF (left) and for the ARH reconstruction (right).

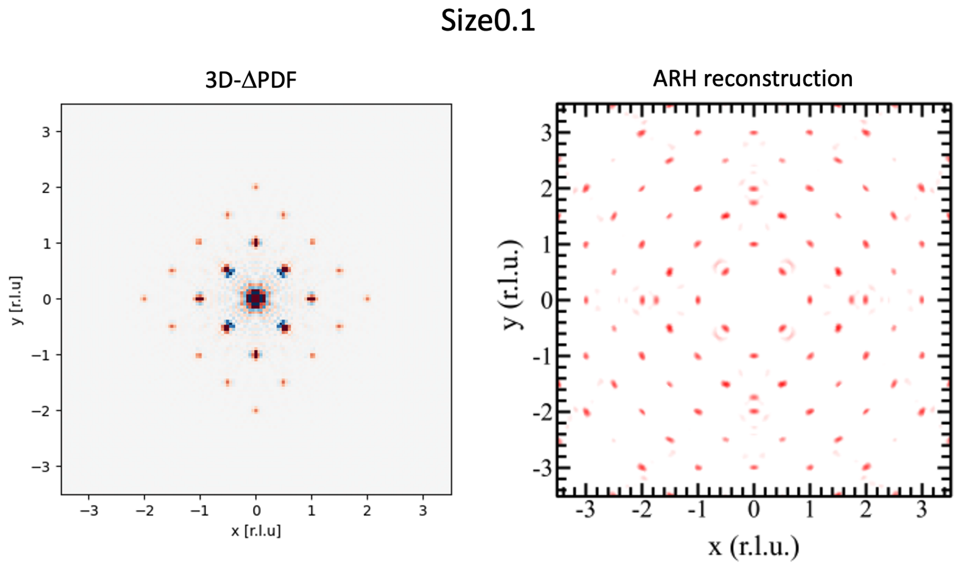

Figure S8: $xy0$-section of the Size0.1 structure for the 3D-ΔPDF (left) and for the ARH reconstruction (right).

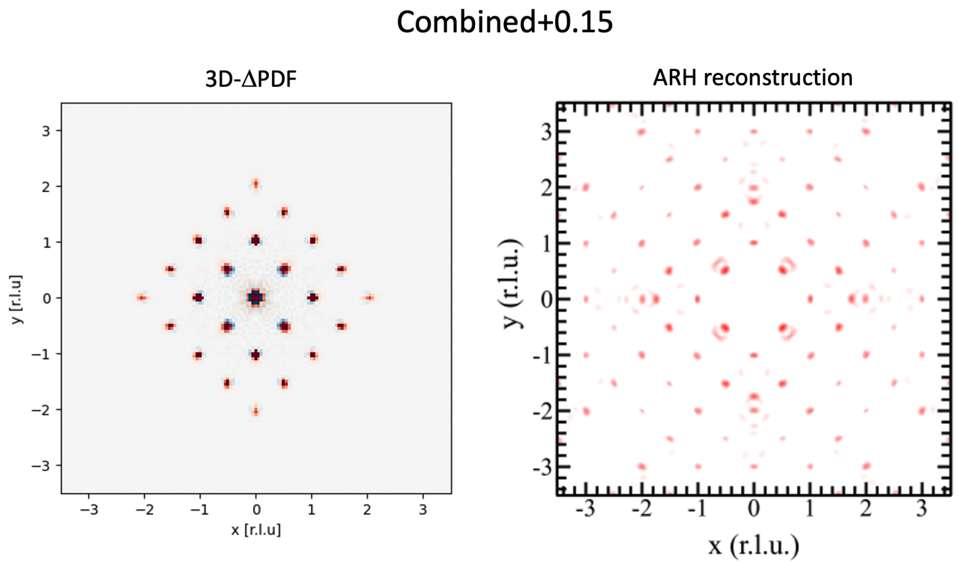

Figure S9: $xy0$-section of the Combined+0.15 structure for the 3D-ΔPDF (left) and for the ARH reconstruction (right).

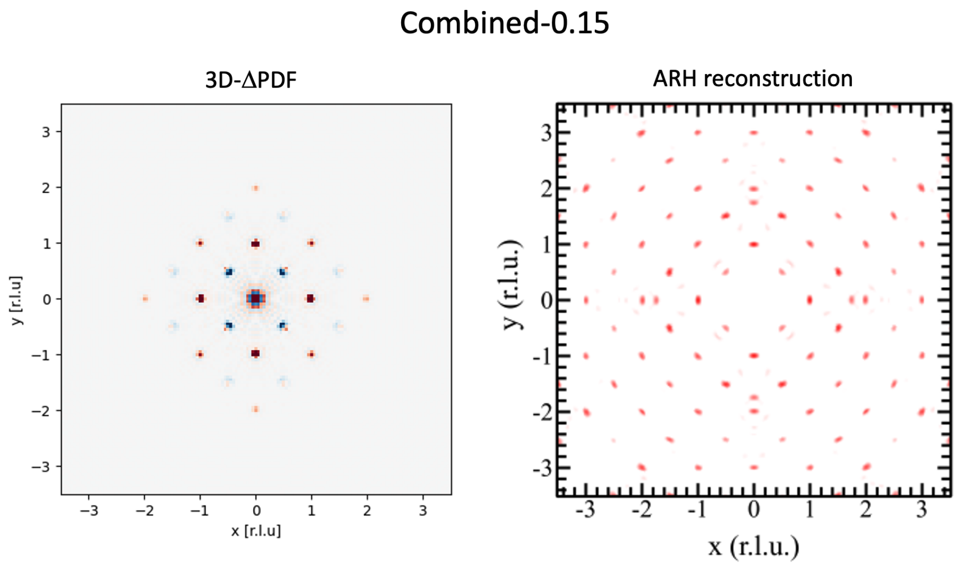

Figure S10: $xy0$-section of the Combined-0.15 structure for the 3D-ΔPDF (left) and for the ARH reconstruction (right).
